# Recovering missing features in nonnegative matrix factorization via generalized singular value decomposition

**DOI:** 10.1016/j.isci.2026.114708

**Published:** 2026-02-12

**Authors:** Youdong Guo, Timothy E. Holy

**Affiliations:** 1 Department of Neuroscience, Washington University in St. Louis, St. Louis, MO, USA; 2 Department of Biomedical Engineering, Washington University in St. Louis, St. Louis, MO, USA

**Keywords:** Applied sciences, Network

## Abstract

Nonnegative matrix factorization (NMF) is widely used to separate mixed sources into components. Algorithms for NMF require choosing the rank in advance, and if the results are unsatisfying, one typically executes NMF again with a different rank. To make NMF more interactive, here we introduce GSVD-NMF, a method that proposes new components based on the generalized singular value decomposition (GSVD) to address discrepancies between initial under-complete NMF results and the SVD of the original matrix. Simulation and experimental results demonstrate that GSVD-NMF often effectively recovers multiple missing components in under-complete NMF, with the recovered NMF solutions frequently reaching better local optima. The results further show that GSVD-NMF is compatible with various NMF algorithms and that directly augmenting components is more efficient than rerunning NMF from scratch with additional components. Furthermore, the under-complete NMF can be computed with a relaxed convergence tolerance, greatly reducing runtime while still enabling accurate feature recovery.

## Highlights

• Recovers missing components from under-complete nonnegative matrix factorization
• Uses generalized singular value decomposition to identify discrepant directions
• Enables efficient rank expansion without rerunning NMF from scratch
• Improves solution quality and efficiency across multiple NMF algorithms

## Graphical abstract

**Figure:**
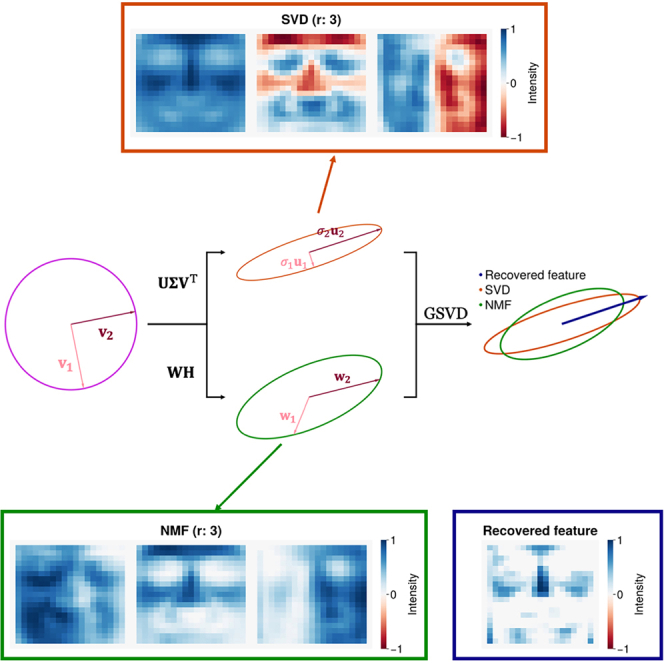

## Introduction

The exponential growth of data, driven by high-throughput technologies, represents a significant opportunity and challenge. To uncover hidden structures within these high-dimensional datasets, nonnegative matrix factorization (NMF) has become a widely-used tool. NMF is an effective unsupervised machine learning approach for extracting latent features in circumstances where plausible features, and their loadings, are constrained to be nonnegative. It has been widely used in the analysis of many types of data.^1^^,^^2^^,^^3^^,^^4^^,^^5^^,^^6^^,^^7^^,^^8^^,^^9^^,^^10^^,^^11^^,^^12^ Compared to other widely used source separation methods, such as PCA and ICA, NMF offers two key advantages: it does not require the assumption that components are uncorrelated or independent, and its results are intuitive and easy to interpret.^13^ Mathematically, an NMF is given by

(Equation 1) $$\begin{array}{c} X\;\approx \;WH\;S.t.\;W\geq 0,\;H\geq 0, \end{array}$$

where $W\in R_{+}^{m\times r}$ and $H\in R_{+}^{r\times n}$ are two nonnegative matrices. r is the number of components, which ideally represents the actual number of features in the data matrix. For example, individual features can refer to attributes such as the pixel-wise intensity of a cell in a microscopic image,^14^ the ion-count vs. mass-to-charge ratio of a compound in a mixed mass profile^15^ or the participation of brain areas in a particular network in cortical functional magnetic resonance imaging.^16^ To obtain W and H in Equation 1, here we focus on NMF without any additional regularization, minimizing the Squared Euclidean Distance (SED) of the original matrix (X) and the factorization (WH)

(Equation 2) $$\begin{array}{c} D_{E}\left(W,H;X\right)=\frac{1}{2}{\left\|X-WH\right\|}_{\text{F}}^{2}, \end{array}$$

where $D_{E}:R_{+}^{m\times r}\times R_{+}^{r\times n}\to R$. The SED is one of the most widely used objective functions for NMF, and a variety of algorithms have been developed to minimize it.^1^^,^^17^^,^^18^^,^^19^

Although NMF is widely used, in some circumstances it can fail to identify an accurate and comprehensive representation of the data features. NMF is NP-hard^15^^,^^20^ and performing an NMF-based analysis requires the determination of a suitable factorization rank. Various methods have been proposed to learn the factorization rank (r), but no known procedure results in an unambiguous answer.^10^^,^^20^^,^^21^ In practice, researchers often explore a range of component numbers to identify the most interpretable or meaningful results.^22^^,^^23^^,^^24^ Moreover, we recognize that in real-world problems, the correct number of components may be undefined, and the usefulness of the decomposition is assessed in part by how well the resulting components explain the underlying phenomena under study. For example, in calcium imaging microscopy, different regions of a cell may exhibit distinct temporal dynamics, and thus a factorization where the number of components equals the number of cells may actually fail to assign one component per cell, if highly-active cells are poorly described with a single component. If changing component number means that NMF has to be restarted from scratch, producing stable and meaningful features can require considerable trial-and-error, and thus be time-consuming for large datasets.

Given that the “true” factorization rank is poorly defined, in this manuscript we focus on a more practical problem: supposing that one decides to change the rank of an existing factorization, how might one do so efficiently? Several methods start with an excess number of components and then prune them. Sparse regression-based NMF pruning removes unnecessary components through sparsity constraints.^25^ These methods do not heavily rely on robust estimation of the number of components, as unnecessary components are effectively reduced to zero. However, they introduce an additional sparsity coefficient, which must be learned or tuned separately. Moreover, different approaches may produce varying results when applied to real-world datasets.^26^^,^^27^^,^^28^ Another approach that relies on starting with excess components was recently developed by Guo et al.,^29^ which progressively merges redundant components to achieve better local optima while dynamically reducing the factorization rank.

The alternative to rank-reduction is rank-expansion, i.e., to begin with a smaller set of components which grows as needed. With an initially undercomplete representation, features may be missed or integrated into other components, so this approach requires the ability to add features in a manner that addresses deficits in the current factorization. Rank-one nonnegative matrix underapproximation^19^^,^^30^ (NMU) incrementally adds one component to an existing W and H, and then re-optimizes the augmented factorization. Supposing $W_{k-1}=\left[w_{1},w_{2},\ldots ,w_{k-1}\right]$ and $H_{k-1}={\left[h_{1},h_{2},\ldots ,h_{k-1}\right]}^{\text{T}}$ satisfy ${\left(W_{k-1}H_{k-1}\right)}_{ij}\leq X_{ij}\forall i,j$, NMU recursively expands the rank by one, adding $w_{k},h_{k}$ minimizing

(Equation 3) $$\begin{array}{c} E_{\text{NMU}}\left(w_{k},h_{k};N_{k}\right)=\|N_{k}-w_{k}h_{k}^{\text{T}}{\|}^{2}\;s.t.\;w_{k}\geq 0,\;h_{k}\geq 0\;and\;w_{k}h_{k}^{\text{T}}\leq N_{k}, \end{array}$$

where $E_{\text{NMU}}\in R_{+}^{m}\times R_{+}^{n}\to R_{+}$ and $N_{k}=X-w_{1}h_{1}^{\text{T}}-\cdots -w_{k-1}h_{k-1}^{\text{T}}$. This rank-expansion is performed for k=1,2,3,…,r. With NMU, the residual $N_{k}$ is required to be nonnegative, which must be enforced as a constraint during optimization.^19^^,^^31^^,^^32^ Because of these constraints and the recursive nature of updating, NMU is computationally expensive, and its convergence remains undemonstrated.^33^

In this paper, we introduce an alternative to NMU called GSVD-NMF, which uses the generalized singular value decomposition (GSVD) to incrementally grow r and augment components for an existing under-complete NMF solution. We use the GSVD to discover the main directions of discrepancy (corresponding to generalized singular values most different from one) between the SVD of the data matrix and the existing NMF of the same rank. As SVD provides a globally optimal matrix factorization, and GSVD identifies conjugate directions that extremize the signal-to-signal ratio, this approach is maximally informative about discrepancies within the subspace spanned by the SVD. We employ a similar truncation as used in NNDSVD^34^ to enforce the nonnegativity of the augmented components.

Compared to NMU, GSVD-NMF introduces two important innovations: (1) We employ GSVD to propose new directions and refine them using standard NMF, providing a deterministic initialization to the missing-component problem. (2) Using nonnegative least squares, we introduce the “new” components while modulating the amplitude of “old” components, thus making room for the new components to contribute substantively to the factorization without any need for constrained optimization.

Although we use the word “incremental” our method is incremental in *rank*, not data: we assume that the underlying reference matrix remains constant. This is different from incremental (online) NMF,^35^^,^^36^^,^^37^^,^^38^^,^^39^^,^^40^^,^^41^^,^^42^^,^^43^^,^^44^^,^^45^ where new data points arrive over time and the model is updated while keeping the rank fixed or adjusting it based on the newly added samples. Given that these are two distinct uses of the word “incremental” we do not compare our method to ones which focus on online data.

The entire pipeline is tested through simulations and on multiple real-world datasets with different NMF algorithms for source separation. The results demonstrate that our method effectively initializes new components for under-complete NMF, with the augmented solutions achieving better local optima and greater efficiency than standard NMF approaches. Moreover, starting from under-complete NMF (deliberately setting rank number $r_{0}$ smaller than the number of features in X), the proposed method can help NMF converge to better local optima. A key practical advantage is that the under-complete NMF can be computed with a relaxed convergence tolerance, greatly reducing runtime while still enabling accurate feature recovery. Finally, the proposed method allows one to efficiently expand the number of components, which can be convenient and effective for interactive analysis of large-scale data.

## Results

The mathematical foundation of GSVD-NMF is described in STAR Methods. In this section, we demonstrate the effectiveness of GSVD-NMF and compare its performance with standard NMF on synthetic and real-world data.

### Concept and illustrative examples

**Figure 1:**
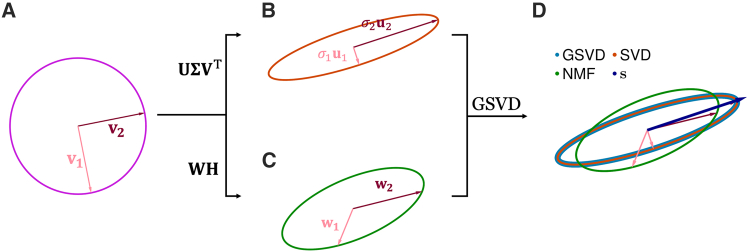
Concept of GSVD-based feature recovery for NMF (2×2 case) (A) A unit disk (magenta) and its basis vectors $v_{1}$, $v_{2}$. (B) Under multiplication by X, points in the magenta disk map to points in the brown ellipse; key elements of X’s SVD are denoted. (C) An inexact NMF factorization maps to a slightly different ellipse (dark-green), indicating an imperfect match to X. (D) GSVD-NMF suggests new directions (s) that maximally capture the discrepancy between the true mapping (SVD ellipse) and the NMF mapping. Under multiplication by $\left[W_{0}diag\left(\alpha \right)\;S\right]\left[H_{0};\;Y\right]$, the magenta disk is mapped to the blue ellipse (GSVD), which aligns with the true SVD ellipse.

Our concept is most easily understood by viewing matrices as linear transformations that act on vectors in the domain space, which in Figure 1 will be represented as points in the unit disc (magenta). Figure 1B represents the action of the SVD of $X=\left(\begin{array}{cc} 3.0 & 0.385\\ 0.84 & 0.8087 \end{array}\right)$ on a unit disc and Figure 1C is action of the NMF of X on the unit disc, projected into the subspace spanned by the SVD. We model an imperfect NMF, given by $W=\left(\begin{array}{cc} 1.5012 & 0.0000\\ 0.2252 & 1.2493 \end{array}\right)$ and $H=\left(\begin{array}{cc} 1.4655 & 0.6395\\ 0.0000 & 0.9605 \end{array}\right)$, which is a relatively poor approximation of our chosen X. This is revealed as a difference between the resulting ellipses in the range-space (Figure 1D). Our primary objective is to identify new directions that can reshape the NMF solution to more closely approximate the SVD solution, as exemplified by the vector s in Figure 1D.

The direction s shown in Figure 1D corresponds to the new column in S produced by solving Equations 5 and 14. Together with adjustments in the amplitudes of the existing components, the augmented solution maps the unit disc onto the blue ellipse (GSVD), perfectly replicating the SVD ellipse (brown). The perfect recovery in this case is an artifact of restricting ourselves to two dimensions for visualization, but it does demonstrate that the GSVD step can perfectly recover the missing feature direction in this example.

**Figure 2:**
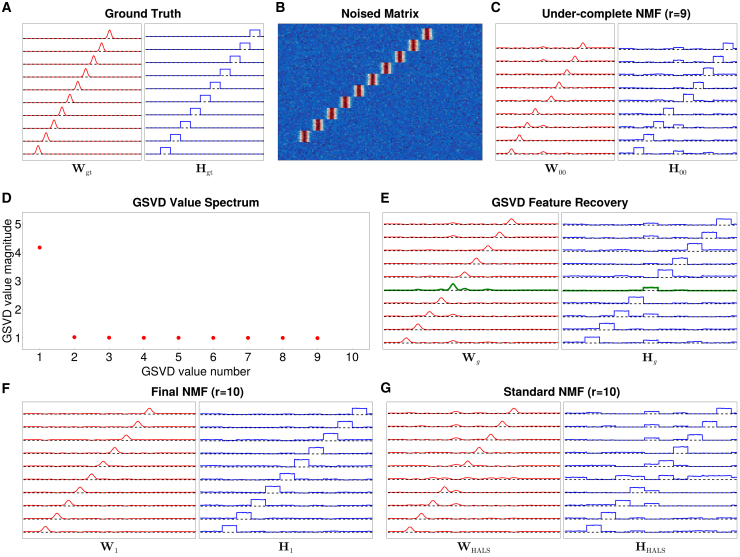
A synthetic example used to illustrate GSVD-NMF for feature recovery (k=1), displaying W and H (A) Ground truth W (each line depicting one column) and H (each line depicting one row) with 10 features. (B) X generated as WH with added Gaussian noise. (C) Standard NMF results (HALS) with 9 components. (D) The generalized singular value spectrum from Equation 7. (E) Feature recovery results ($W_{g}$, $H_{g}$), with the new component in green. (F) Final NMF results ($W_{1}$, $H_{1}$). (G) Standard NMF results initialized with NNDSVD. Despite knowing the correct number of components, several features are incompletely separated, and the solution is much worse than (F).

For a slightly more realistic example, we illustrate the ability of GSVD-NMF to discover “missing” components on synthetic data with 10 ground truth components, which are shown in Figure 2A. Running HALS,^17^ one of the most accurate and widely-used NMF algorithms,^20^^,^^46^ with 10 components initialized using NNDSVD,^34^ on X=WH with Gaussian noise (as shown in Figure 2B) resulted in an inaccurate solution (Figure 2G). The solution included multiple feature components and a noise component, with one feature failing to capture an independent component.

To exploit GSVD-NMF, we started from the solution returned by HALS with 9 components, one less than the number of ground truth components, obtaining the result shown in Figure 2C. One sees that several components of the ground truth are blended, Figure 2C. To test for missing information, we compared the 9-component NMF with a 9-component SVD using GSVD as described in STAR Methods. This identified a “missing” direction related to the first (large-magnitude) generalized singular value, Figure 2D. Note that this analysis provides support for only one additional component. The component recovered by the feature recovery step in the pipeline (Figure S1) is shown in green in Figure 2E. After using HALS to optimize this 10-component augmented factorization, the final NMF result, shown in Figure 2F, faithfully represents each ground truth component. Therefore, GSVD-NMF can recover the missing component from under-complete NMF, and in this case performs better than naive HALS even when the correct number of components is known and a high-quality initialization strategy is used.

**Figure 3:**
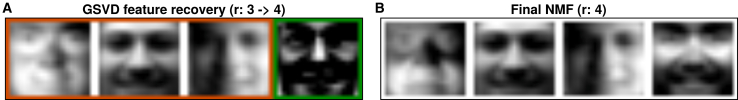
GSVD-NMF feature recovery illustrated on the CBCL face image dataset (W component) (A) A rank-3 NMF is augmented with one additional component (green box), while scaling the old components (orange box) by α (Equation 13). (B) Final NMF result obtained by refining the initialization in (A).

For an even more realistic example, Figure 3 illustrates the process of adding a single component to a rank-3 NMF of the CBCL face image dataset. The new component (green box, panel (a)) captures meaningful facial structures (principally, the nose and cheeks) that were under-emphasized in the rank-3 NMF solution. Subsequent NMF refinement (panel (b)) redistributes features among the four components, while maintaining an obvious connection to the initial state in panel (a). This example clearly demonstrates how GSVD-NMF can reveal and separate features that standard NMF fails to distinguish at lower ranks.

### Experimental results

#### Datasets

**Figure 4:**
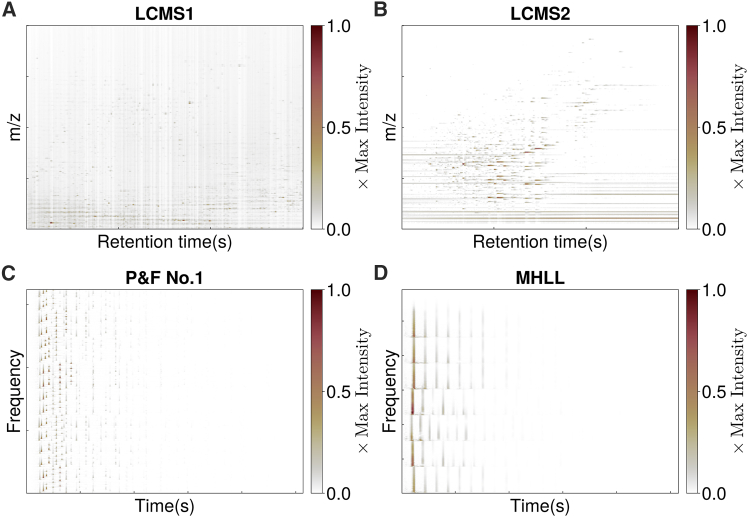
Four of the real-world datasets used for experiments (A) LCMS1. (B) LCMS2. (C) The amplitude spectrogram of “prelude and fugue no.1 in C major”. (D) The amplitude spectrogram of “Mary had a little lamb.” The colorbar label represents the intensity at each pixel, normalized by the maximum intensity of the matrix. For the LCMS data, the intensity corresponds to the ion count. Higher values indicate a greater number of ions.

We also tested the performance of GSVD-NMF on seven real-world datasets: two liquid chromatography mass spectrometry (LCMS) datasets, three face image datasets, and two audio datasets. The LCMS datasets are shown in Figures 4A and 4B. Two of the face image datasets, the MIT CBCL face images^47^ and the ORL face images from AT&T Laboratories Cambridge, were analyzed systematically using the same procedures as for the LCMS and audio datasets. The audio datasets feature the first 30 s of “Prelude and Fugue No.1 in C major” by J.S. Bach, played by Glenn Gould and the first measure of “Mary had a little lamb”, both of which are taken ifrom Leplat et al., Cichocki and Phan, Lin^8^^,^^17^^,^^18^ and illustrated in Figures 4C and 4D. The third face image dataset, Extended YaleB face images, is used as an example of “big data”. To demonstrate the computational advantages of GSVD-NMF; for this dataset, we only report results with deterministic initialization and compare the resulting convergence trajectories.

Details about all seven real-world datasets are presented in Table 1 and the column r lists the “real” number of features we selected for this paper. Where possible, these choices were based on previous literature with the same datasets.^8^ For the LCMS datasets not previously studied, we tested several rank-selection methods.^10^^,^^20^^,^^48^ Because these methods gave divergent answers, ultimately we selected one among the recommended values based on visual inspection.

**Table 1:** Description of datasets

No.	Datasets	Size	r
1	LCMS1	600×400	17
2	LCMS2	600×400	23
3	MIT CBCL face images	361×2429	49
4	ORL face images	10304×400	25
5	Prelude and Fugue No.1 in C major (P&F No.1)	647×513	13
6	Mary had a little lamb (MHLL)	294×257	3
7	Extended YaleB face images (large matrix test)	32256×2424	28, 32, 64, 128

#### GSVD-NMF recovers components for under-complete NMF

While the illustrations above used a direct approach—solving Equations 5 and 14—to initialize new rows in H and columns in W, this approach was computationally expensive on larger datasets. Henceforth, we adopt an alternative accelerated initialization for W: starting with Equation 16 and following the pipeline shown in Figure S1).

**Figure 5:**
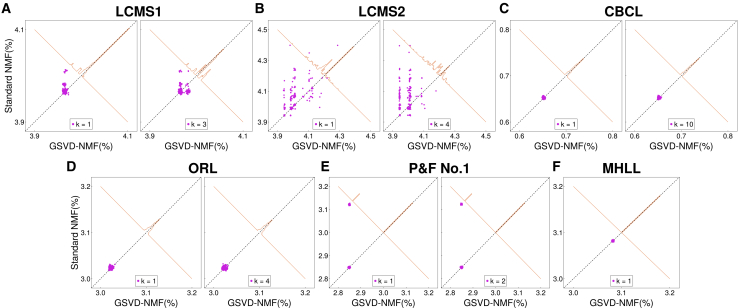
Comparing the fitting error of standard NMF (HALS) and GSVD-NMF on real-world data (A–F) Left and right panels corresponds to a different number of components recovered by GSVD-NMF. (A) LCMS1. (B) LCMS2. (C) CBCL. (D) ORL. (E) prelude and fugue no.1 in C major. (F) Mary had a little lamb. The scatterplot compares the relative fitting errors of standard NMF and GSVD-NMF against the original matrix across different NMF algorithms. Each magenta scatter point represents an individual comparison from a single random initialization, with its position indicating the relative fitting error for standard NMF (vertical axis) and GSVD-NMF (horizontal axis). The brown line illustrates the histogram of the perpendicular distances from the scatter points to the diagonal, summarizing the overall distribution of error differences. For most tests, GSVD-NMF produces an equal or better fit.

The comparison of local optima achieved by standard NMF and GSVD-NMF is given in Figure 5 (comparison with HALS) and Figure S2 (comparison with (GCD, ALSGrad, and MU) in the supplementary materials. Here, we show the results for k=1 (a single new component) and $k=0.2r_{0}$ (recovering 20% of the number of components in the under-complete NMF). Both are evaluated via relative fitting error, with the horizontal axis representing GSVD-NMF and the vertical axis standard NMF.

Figures 5 and demonstrate that NMF converges to single or multiple stationary points across all datasets using all four algorithms. Most are local minima, but for the MU algorithm some clusters in Figure S2C contained solutions that could be substantially improved via HALS; since HALS optimizes Equation 2 strictly by descent, these MU-solutions did not lie at the bottom of a local basin. For all algorithms, most points in Figures 5 and are above or along the diagonal line, indicating that the recovered solution from under-complete NMF by GSVD-NMF either matches or improves the convergence of NMF in the majority of cases. This conclusion was true both when adding a single component (k=1) or when adding multiple components simultaneously $\left(k=0.2r_{0}\right)$.

**Figure 6:**
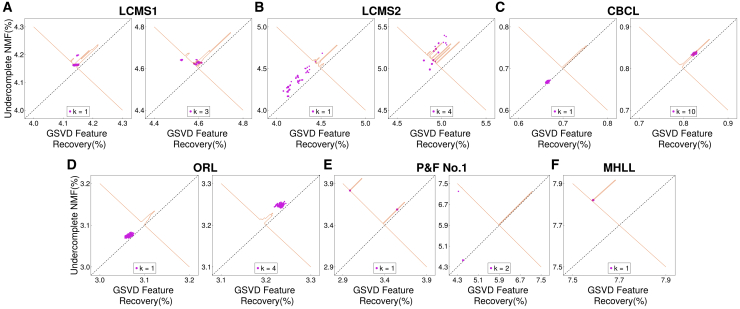
Improvement in objective value using the GSVD-NMF features Left and right panels corresponds to a different number of components recovered by GSVD-NMF (A) LCMS1. (B) LCMS2. (C) CBCL. (D) ORL. (E) Prelude and fugue no.1 in C major. (F) Mary had a little lamb.

Although we show in the STAR Methods section that the fitting error cannot increase after inserting new components—because the under-complete NMF solution is always embedded within the augmented solution—we empirically verify this property here. The scatterplots in Figures 6 and S3 (supplemental information) compare the fitting errors of the re-initialized solutions (after component insertion but before NMF refinement) with those of the under-complete NMF results. Empirically, the figure shows that GSVD-based component injection never increases the fitting error, and subsequent NMF refinements converge quickly from these initializations. Although the improvement can be subtle in some datasets, the re-initialized solutions consistently achieve equal or lower error than the under-complete NMF, as indicated by points lying above the diagonal (e.g., the case k=2 for “Prelude and Fugue No. 1 in C major”). This confirms that no case arises in which all newly added components collapse to zero.

We also evaluated whether GSVD-NMF could achieve gains over existing approaches when using deterministic initialization methods such as NNDSVD, NNDSVDa, and NNDSVDar.^34^ The results are shown in Tables 2 and S1. The NMF solutions recovered by GSVD-NMF are comparable to or better than standard NMF across all datasets and all tested NMF algorithms, whether recovering single or multiple components simultaneously. This outcome demonstrates the perhaps surprising point that GSVD-NMF can improve NMF even when the same SVD components have already been used to initialize NMF.

**Table 2:** GSVD-NMF vs. standard NMF (HALS) with deterministic initialization

Datasets	Fitting error (%): Standard NMF/GSVD-NMF					
	r	k	Random	NNDSVD	NNDSVDa	NNDSVDar
LCMS1	17	1	3.97±0.01/3.97±0.00	3.97/3.97	3.97/3.97	3.97/3.97
		3	3.97±0.01/3.97±0.01	3.97/3.97	3.97/3.97	3.97/3.97
LCMS2	23	1	**4.06**±**0.08/4.01**±**0.06**	**4.05/3.99**	**4.07/4.01**	**4.05/3.99**
		4	**4.06**±**0.08/3.99**±**0.04**	**4.05/4.01**	**4.07/4.01**	**4.05/4.01**
CBCL	49	1	0.65±0.00/0.65±0.00	0.65/0.65	0.65/0.65	0.66/0.65
		10	0.65±0.00/0.65±0.00	0.65/0.65	0.65/0.65	0.66/0.65
ORL	25	1	3.02±0.00/3.02±0.00	3.02/3.02	3.03/3.02	3.02/3.02
		4	3.02±0.00/3.02±0.00	3.02/3.02	3.03/3.02	3.03/3.02
P&F No.1	13	1	**2.90**±**0.11/2.85**±**0.00**	2.85/2.85	2.85/2.85	2.85/2.85
		4	**2.90**±**0.11/2.85**±**0.00**	2.85/2.85	2.85/2.85	2.85/2.85
MHLL	3	1	3.08±0.00/3.08±0.00	3.08/3.08	3.08/3.08	3.08/3.08

Bold entries indicate lower fitting error for GSVD-NMF than standard NMF.

**Figure 7:**
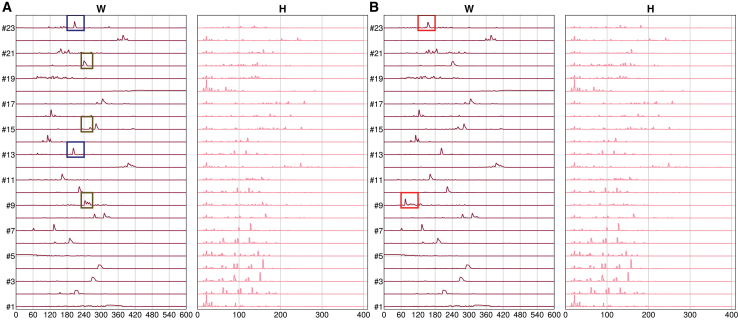
Comparison of the factorized components (W, H) obtained from NMF on LCMS2 data using HALS with NNDSVD initialization The red boxes highlights components captured by GSVD-NMF but missed by standard NMF. The blue and brown boxes indicate a single feature that is split across multiple components in the standard NMF results, whereas GSVD-NMF successfully captures it as a single component. (A) Standard NMF. (B) GSVD-NMF.

Even when the improvement in relative fitting error over HALS may seem modest (e.g., approximately 3% on LCMS2), it results in substantial differences in the extracted components. Since obtaining these components is typically the actual goal of performing NMF, such improvements may have substantial impact on downstream analyses. Figure 7 presents a comparison between the components derived from standard NMF (HALS) and GSVD-NMF (performed in the recursive way) on the LCMS2 dataset. Specifically, components #9 and #23 (marked with green boxes in Figure 7B) are clearly recovered by GSVD-NMF but are missing in the HALS results. In the HALS decomposition, the features corresponding to component #9 are fragmented across components #20, #15, and #3 (blue boxes in Figure 7A), while GSVD-NMF preserves these components individually. Likewise, component #23 is entangled with component #13 in the HALS output, whereas GSVD-NMF distinguishes #13 as an independent feature (brown box in Figure 7A).

#### Comparison to nonnegative matrix underapproximation (NMU)

As mentioned in the introduction, GSVD-NMF can be compared to NMU, which also iteratively grows the rank of the factorization. To ensure a fair comparison, we started GSVD-NMF with two components and incrementally added one component at each step, matching the sequential nature of NMU. The results are summarized in Table 3, with the full error trajectories versus the number of components shown in Figure S5 of the Supplementary Materials. These trajectories indicate that GSVD-NMF consistently maintains a lower fitting error as the rank increases. GSVD-NMF outperforms all NMU algorithms on all datasets, and matches or beats HALS on all datasets. We suspect NMU’s worse performance on Equation 2 is due to the additional constraint WH≤X, which may restrict its optimization flexibility.

**Table 3:** GSVD-NMF vs. nonnegative matrix underapproximation

Datasets	Fitting error (%)				
	RNMU	LNMU	PNMU	NMU-ADMM	GSVD-NMF
LCMS1	6.14	5.81	6.03	8.55	3.97
LCMS2	6.21	6.66	6.47	10.72	3.94
CBCL	3.03	1.66	2.37	4.09	0.65
ORL	5.08	6.66	4.53	13.56	3.02
P&F No.1	4.87	5.37	4.79	8.89	2.85
MHLL	6.11	5.60	6.30	10.29	3.08

### Computational performance: GSVD-NMF finds high-quality solutions more quickly

Aside from the quality of solutions, many applications of NMF are sensitive to computational performance. Superficially, one might imagine that GSVD-NMF would increase the computational cost, given the extra steps that include additional optimization runs. To investigate this issue, we measured the time required to augment an existing under-complete solution and compared it to the time required to run NMF again with more components. Specifically, starting from the same initialization we performed NMF twice: once to measure the average time per iteration (which varies with dataset and number of retained components), and a second time to measure the objective value after each iteration until convergence. Measuring these two quantities separately was necessary because computation of the objective value takes much longer than a single iteration of NMF.

**Figure 8:**
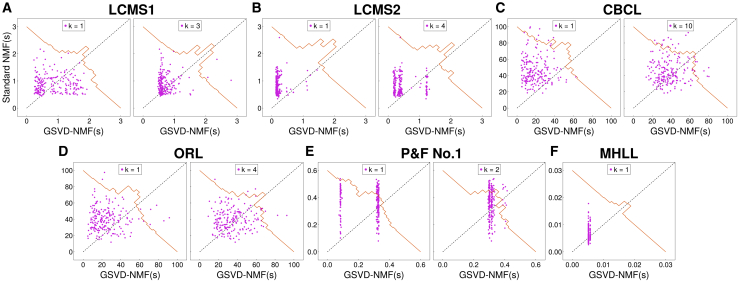
Total runtimes of standard NMF vs. GSVD-NMF with different random initializations (A–F) Each point represents the runtime for standard NMF plotted against the runtime for GSVD-NMF; points above the diagonal indicate shorter runtime for GSVD-NMF. The brown line shows the histogram of the perpendicular distances from the diagonal, summarizing the overall distribution of time differences. (A) LCMS1. (B) LCMS2. (C) CBCL. (D) ORL. (E) Prelude and fugue no.1 in C major. (F) Mary had a little lamb. For most datasets, GSVD-NMF converged to high-quality solutions more quickly.

The results for random initialization are presented in Figure 8, as a scatterplot comparing the time required to augment components in under-complete NMF by GSVD-NMF versus the time to run NMF from scratch with additional components. The title of each panel within the subplot provides the name of the dataset. Similar to Figure 5, each dot represents a single NMF run with a specific initialization (now measuring run-time rather than objective value), while the brown lines indicate the distribution of distances between each dot and the diagonal. Figure 8 shows that in most cases, initializing new components by GSVD-NMF is generally more efficient than rerunning NMF with extra components. As suggested by the thin vertical clusters that frequently appear in these plots, the time taken for GSVD-NMF augmentation was relatively consistent for each under-complete local minimum, while the times for running NMF from scratch with additional random components varied significantly. The CBCL and ORL face image datasets provide an exception: their GSVD-NMF runtimes do not form tight vertical clusters. This is because the under-complete NMF solutions for these datasets contain many distinct local optima with similar fitting errors, as noted in the study by Guo et al.^29^ Nevertheless, even in these cases, GSVD-NMF remains more efficient than rerunning NMF from scratch, as indicated by the fact that more points lie above the diagonal.

For deterministic initializations (SVD-based initialization), Figure S4 in the Supplementary materials show the traces of objective value versus time for different initialization methods across four datasets. Each panel’s caption includes the dataset name and the number of new components added. The GSVD-NMF trace represents the final NMF phase used for refinement. The first element in each trace represents the objective value after one iteration. Figure S4 indicates that the final NMF in GSVD-NMF converges faster than standard NMF for both LCMS datasets, face image datasets and “Mary had a little lamb.” In contrast, for “prelude and fugue no.1 in C major,” the convergence performance is similar to or slightly worse than that of standard NMF.

Further zooming into the region around the intersection of the two strategies’ traces reveals that for k=1, the proposed method behaves similarly to standard NMF. However, for k=2, it achieves a slightly lower objective value after a period of slow progress, indicating a minor advantage in the final objective, albeit with slower convergence.

In conclusion, we find that it is generally more efficient to augment a prior solution by GSVD-NMF than it is to run standard NMF from scratch with additional components. Thus, on average, GSVD-NMF improves upon both quality and computational performance.

The performance of each stage in GSVD-NMF is documented in Table 4. It is evident that the “feature recovery” step is highly efficient, representing an essentially negligible portion of the total time across all datasets. Therefore, the efficiency of GSVD-NMF primarily depends on the time allocated to the NMF step in the pipeline. In other words, ϵ (or the maximum number of iterations) serves as the main control parameter for the runtime performance of GSVD-NMF.

**Table 4:** Time (mean ± std) of each stage of GSVD-NMF

Datasets	Time(s)				
	r	k	Feature Recovery	Final NMF	Standard NMF
LCMS1	17	1	**0.0015**±**0.0001**	0.8561 ± 0.4939	0.9039 ± 0.3380
		3	**0.0013**±**0.0001**	0.7209 ± 0.2950	0.9039 ± 0.3380
LCMS2	23	1	**0.0020**±**0.0002**	0.2220 ± 0.1924	0.8355 ± 0.3179
		4	**0.0018**±**0.0002**	0.4750 ± 0.3691	0.8355 ± 0.3179
CBCL	49	1	**0.0338**±**0.0102**	19.6774 ± 10.9766	46.3339 ± 16.4238
		10	**0.0734**±**0.0871**	37.9356 ± 13.4198	46.3339 ± 16.4238
ORL	25	1	**0.3322**±**0.1677**	24.5752 ± 14.9768	40.0731 ± 13.9827
		4	**0.4761**±**0.1858**	34.7749 ± 14.9396	40.0731 ± 13.9827
P&F No.1	13	1	**0.0011**±**0.0001**	0.2461 ± 0.1133	0.3616 ± 0.1014
		2	**0.0015**±**0.0001**	0.3125 ± 0.0229	0.3616 ± 0.1014
MHLL	3	1	**0.0001**±**0.0000**	0.0052 ± 0.0003	0.0063 ± 0.0024

Bold entries indicate that the GSVD feature recovery step requires substantially less time than the NMF stage.

#### The choice of $\epsilon_{0}$

A potential additional optimization is based on the observation that GSVD-NMF runs standard NMF twice: once for the initial under-complete NMF and again for the final NMF (Figure S1). So far, we employed the same convergence criterion, $\epsilon =10^{-4}$ in Equation 21, for both NMF runs. Here, we investigate whether one can reduce computation time by relaxing the tolerance of the initial under-complete NMF without sacrificing overall quality.

**Figure 9:**
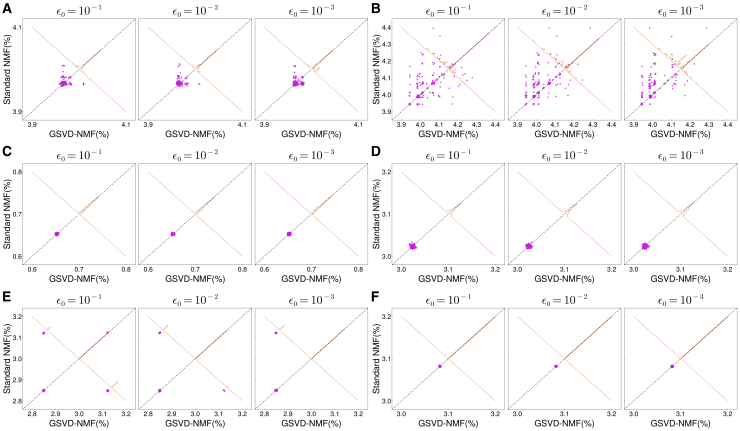
The effect of convergence tolerance $\epsilon_{0}$ on final results of GSVD-NMF (A–F) Panels should be compared to those of Figures 5 and , which used $\epsilon_{0}=10^{-4}$. (A) LCMS1. (B) LCMS2. (C) CBCL. (D) ORL. (E) Prelude and fugue no.1 in C major. (F) Mary had a little lamb.

The effects on solution quality of reducing the stringency of the under-complete NMF $\left(\epsilon_{0}\right)$ steps are shown in Figure 9. Here, we present the results from HALS for NMF with k=1. The tolerance had little effect on the “Mary had a little lamb” dataset. For the other three datasets, the advantages of GSVD-NMF increase with the stringency of $\epsilon_{0}$, particularly evident in the “prelude and fugue no.1 in C major” dataset. Overall, these results demonstrate that in GSVD-NMF, the under-complete NMF can be run at higher tolerance (lower stringency) but with higher risk of landing in worse local optima. However, at all tested stringencies GSVD-NMF outperforms or matches standard NMF in aggregate. Therefore, based on the results of this section and Figure 5, GSVD-NMF could be considered a general pipeline for performing NMF.

#### The choice of k

It should be noted that all of the above experiments set k=1 or $k=0.2r_{0}$, both of which appear to be broadly successful. In practice, we may wish to add more components simultaneously, rather than iteratively adding one component at a time, to make the whole pipeline more efficient. Thus, it is valuable to investigate the role of k on the final results.

**Figure 10:**
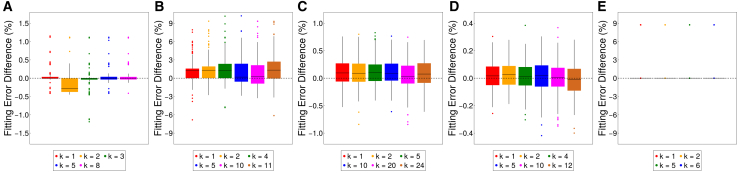
The effect of different k (A) LCMS1. (B) LCMS2. (C) CBCL. (D) ORL. (E) Prelude and fugue no.1 in C major. This figure compares the difference in relative fitting error between GSVD-NMF and standard NMF (${Error}_{\text{Standard}\;\text{NMF}}$-${Error}_{\text{GSVD}-\text{NMF}}$) across different numbers of augmented components. The boxplot summarizes the distribution of fitting error differences for each case. The box spans the interquartile range (IQR), with the midline marking the median, and the whiskers extending to 1.5×IQR.a Positive values indicate that GSVD-NMF achieves a better fit, while negative values suggest that standard NMF performs better.

The experiment for Figure 5 (200 random initialized trials) was conducted with different k. Figure 10 illustrates the difference between the fitting error of standard NMF and that of GSVD-NMF. Consistent with the results in Figure 5, GSVD-NMF finds superior local optimum in most cases on both LCMS datasets and “prelude and fugue no.1 in C major” regardless of k. Thus, the choice of k is not critical, as a wide range of choices (at least for fairly small values of k) work well.

### Application on large-scale matrix

**Figure 11:**
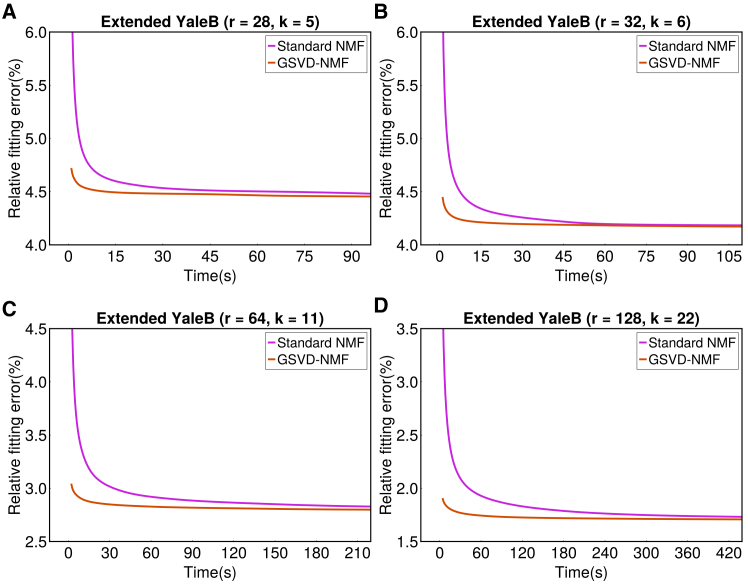
Runtimes of standard NMF and GSVD-NMF with NNDSVD initialization on the extended YaleB dataset (large matrix) The plot shows convergence trajectories (objective value vs. time), demonstrating that GSVD-NMF converges faster than re-running standard NMF from scratch.

To further demonstrate the computational advantage of expanding rank via GSVD-NMF rather than re-running NMF from scratch, we conducted an additional experiment on a genuinely large matrix: one pose from the Extended YaleB face dataset (#7 in Table 1), with size 32256×2424 (approximately 78 million entries). We tested several target ranks (28,32,64,128) to provide a comprehensive comparison across different factorization sizes. Figure 11 reports the convergence trajectories (objective value vs. time) for GSVD-NMF and standard NMF (300 iterations). The runtime of the GSVD feature-recovery step is included, which shifts the GSVD-NMF curves slightly to the right; however, this overhead corresponds to only one or two NMF iterations (as shown in the Methods section) and is negligible relative to the full optimization. Across all tested ranks, GSVD-NMF converges substantially faster than re-running standard NMF from scratch, and the advantage becomes more pronounced at higher ranks. Overall, the large-scale results are fully consistent with our findings on small and medium-sized datasets: augmenting a prior solution with GSVD-NMF is more efficient than restarting NMF with a larger rank, especially on large matrices where re-initializing and re-optimizing from scratch is costly.

## Discussion

In this work, we introduced GSVD-NMF, a framework for recovering missing features in under-complete NMF. By leveraging the GSVD between the under-complete NMF result and the SVD of the original data, the method identifies latent subspace directions that the under-complete NMF fails to capture and incrementally augments the components to recover these missing features. Experiments on synthetic and real-world datasets demonstrate that GSVD-NMF effectively restores omitted features, achieves comparable or lower reconstruction error than re-running NMF from scratch, and remains robust across a range of added component numbers. Expanding the rank with GSVD-NMF is therefore substantially more efficient than performing a full NMF recomputation. A further practical advantage is its tolerance to relaxed convergence in the initial under-complete NMF. Because GSVD-NMF depends primarily on the subspace structure rather than exact coefficient estimates, the initial factorization can be computed with a loose tolerance, greatly reducing runtime without compromising recovery accuracy. This property makes the method both efficient and flexible for large-scale or exploratory analyses where rank selection and full convergence are expensive. Overall, GSVD-NMF provides an effective and principled mechanism for incrementally expanding the rank of NMF solutions, bridging SVD-based subspace analysis with NMF. It offers a practical route toward adaptive rank growth, improved interpretability, and enhanced computational efficiency in large-scale matrix factorization problems.

### Limitations of the study

Our work demonstrates the advantages of GSVD-NMF for adding new components. However, several points remain worthy of further investigation.

First, in this work we used a simplistic criterion for choosing how many components to add, e.g., adding 20% additional components. One promising enhancement might be to monitor the generalized singular value spectrum and identify an “elbow” point that indicates diminishing returns in added components.

Second, as described in the STAR Methods section, the procedures “init H” and “init W″ do not guarantee nonnegativity of the newly added rows and columns in H and W. To address this, the NNDSVD truncation is applied to enforce nonnegativity of the new components. Developing alternative high-quality truncation strategies, or extending the current approach to ensure nonnegativity more directly, represents a promising direction for future work. One possible strategy is to retain both the positive and negative parts during the NNDSVD truncation stage, re-initialize 2k intermediate components, and subsequently apply the component-merging procedure proposed in^29^ to reduce them to k effective components.

Third, the current formulation assumes continuous-valued input matrices and optimizes the fitting under the SED. For discrete data types, such as count matrices (common in text mining or genomics) or binary matrices (e.g., presence/absence data), the SED may not accurately reflect the underlying statistical noise model. In principle, the GSVD-based feature recovery framework could be extended to these settings by incorporating appropriate objective functions—such as KL divergence objectives—within the same rank-expansion scheme. Investigating such extensions of GSVD-NMF may be another important direction for future research.

## Resource availability

### Lead contact

Requests for further information and resources should be directed to and will be fulfilled by the lead contact, Youdong Guo (youdong.guo@wustl.edu).

### Materials availability

Not applicable.

### Data and code availability

• Code: all code required for this paper is open-source and available at https://github.com/HolyLab/GsvdInitialization.jl.
• Data: The datasets analyzed during the current study are available in the Zenodo repository, https://doi.org/10.5281/zenodo.17844816. Original source: LCMS1: available at massIVE: dataset identifiers MSV000089200 and https://doi.org/10.25345/C5KP7TV9T. LCMS2: available at massIVE: dataset identifiers MSV000089747 and https://doi.org/10.25345/C58C9R77T.
• All other items: Any additional information required to reanalyze the data reported in this paper is available from the lead contact upon request.

## Acknowledgments

This work was supported by 10.13039/100000002NIH grants R01DC020034 and R01DC010381 (T.E.H.), and Imaging Science Pathway Fellowship (Y.G., PI: Joseph P. Culver).

## Author contributions

Y.G. and T.E.H. developed the method, Y.G. conducted the experiments. Y.G. and T.E.H. analyzed the results. All authors reviewed the manuscript.

## Declaration of interests

The authors declare no competing interests.

## Declaration of generative AI and AI-assisted technologies in the writing process

During the preparation of this work, ChatGPT was used to refine English usage. After using this tool, the authors reviewed and edited the content and take full responsibility for the content of the publication.

## STAR★Methods

### Key resources table

**Table:** 

REAGENT or RESOURCE	SOURCE	IDENTIFIER
**Deposited data**		

The datasets used for benchmark	https://doi.org/10.5281/zenodo.17844816	LCMS1, LCMS2, CBCL, ORL, Maryhadalittlelamb, Prelude, CroppedYaleB, RandomSeeds

**Software and algorithms**		

Julia 1.10.8	https://julialang.org/downloads/oldreleases/	Programing language
GsvdInitialization.jl	https://github.com/HolyLab/GsvdInitialization.jl	Julia code
NMF.jl	https://github.com/JuliaStats/NMF.jl	Julia code
TSVD.jl	https://github.com/JuliaLinearAlgebra/TSVD.jl	Julia code
LinearAlgebra.jl	https://github.com/JuliaLang/LinearAlgebra.jl	Julia code

### Experimental model and study participant details

Omitted as our study does not involve biological models.

### Method details

#### GSVD-based feature recovery

GSVD has been used to solve the quadratic optimization problem in maximum signal fraction analysis (MSFA),^49^^,^^50^^,^^51^^,^^52^ which seeks a set of projection directions ω that maximize the ratio of signal energy to noise energy, formulated as the Rayleigh quotient

(Equation 4) $$\begin{array}{c} \underset{\omega \neq 0}{\max}J\left(\omega \right)=\frac{\|X\omega {\|}^{2}}{\|N\omega {\|}^{2}}, \end{array}$$

where $X\in R^{m\times n}$ is the observed data matrix. The matrix N approximates the noise component under the additive model $X=X^{*}+N$, with $X^{*}\in R^{m\times n}$ representing the unobserved source signal. Although MSFA does not guarantee exact recovery of the sources, it linearly projects the observed signal onto directions that emphasize components with high signal-to-noise ratio. A theoretically equivalent solution to the optimization problem can be obtained by computing the GSVD between X and N.

Given that $U_{r_{0}}\Sigma_{r_{0}}V_{r_{0}}^{\text{T}}\;\approx \;W_{0}H_{0}+SY$, where S and Y denote the components wait to be recovered, the GSVD of a pair of matrices identifies conjugate directions that maximize the oriented signal-to-signal ratio. Naively, similar to maximizing variance for principal component analysis (PCA), one might imagine that the new direction $y\in R^{n}$ could be obtained by maximizing

(Equation 5) $$\begin{array}{c} E\left(y;U_{r_{0}},\Sigma_{r_{0}},V_{r_{0}},W_{0},H_{0}\right)=\frac{\|U_{r_{0}}\Sigma_{r_{0}}V_{r_{0}}^{\text{T}}y{\|}^{2}}{\|W_{0}H_{0}y{\|}^{2}}, \end{array}$$

where $E:R^{n}\to R_{+}$. Equation 5 formalizes the process illustrated in Figure 1, the generalized Rayleigh quotient quantifies how much residual variance remains outside the NMF subspace, and its maximization (s) effectively “reshapes” the current factorization toward the missing directions. We model y in the column space of V and instead optimize a variant of Equation 5 that projects the NMF result down to the SVD subspace:

(Equation 6) $$\begin{array}{c} \hat{E}\left(\hat{y};U_{r_{0}},\Sigma_{r_{0}},V_{r_{0}},W_{0},H_{0}\right)=\frac{\|\Sigma_{r_{0}}\hat{y}{\|}^{2}}{\|U_{r_{0}}^{\text{T}}W_{0}H_{0}V_{r_{0}}\hat{y}{\|}^{2}}, \end{array}$$

where $\hat{y}\in R^{r_{0}}$, $y=V_{r_{0}}\hat{y}$ and $\hat{E}:R^{n}\to R_{+}$. This is equivalent to optimizing the generalized Rayleigh Quotient. Since $\Sigma_{r_{0}}^{\text{T}}\Sigma_{r_{0}}$ and $V_{r_{0}}^{\text{T}}H_{0}^{\text{T}}W_{0}^{\text{T}}U_{r_{0}}U_{r_{0}}^{\text{T}}W_{0}H_{0}V_{r_{0}}$ are symmetric, the extreme values of Equation 6 satisfy a generalized eigenvalue problem^53^

(Equation 7) $$\begin{array}{c} \Sigma_{r_{0}}^{\text{T}}\Sigma_{r_{0}}\hat{y}=\lambda V_{r_{0}}^{\text{T}}B^{\text{T}}U_{r_{0}}U_{r_{0}}^{\text{T}}BV_{r_{0}}\hat{y}, \end{array}$$

where $B=W_{0}H_{0}$. Equation 7 can be solved to higher numerical precision by the GSVD of $\Sigma_{r_{0}}$ and $U_{r_{0}}^{\text{T}}BV_{r_{0}}$, resulting in

(Equation 8) $$\begin{array}{cc} & \Sigma_{r_{0}}=M_{1}D_{1}Q^{\text{T}},\\ & U_{r_{0}}^{\text{T}}BV_{r_{0}}=M_{2}D_{2}Q^{\text{T}}. \end{array}$$

The matrices $M_{1},M_{2}\in R^{r_{0}\times r_{0}}$ are unitary and $Q\in R^{r_{0}\times r_{0}}$. Here, the matrices $D_{1}\in R_{+}^{r_{0}\times r_{0}}$ and $D_{2}\in R_{+}^{r_{0}\times r_{0}}$ are given by

(Equation 9) $$\begin{array}{c} D_{1}=\left(\begin{array}{cc} I & 0\\ 0 & C \end{array}\right),\;\;D_{2}=\left(\begin{array}{cc} 0 & G\\ 0 & 0 \end{array}\right), \end{array}$$

where $C,G\in R^{l\times l}$ are real, diagonal matrices and l denotes the rank of $U_{r_{0}}^{\text{T}}BV_{r_{0}}$. $I\in R_{+}^{\left(r_{0}-l\right)\times \left(r_{0}-l\right)}$ is identity matrix. Plugging Equation 8 into Equation 7 yields

(Equation 10) $$\begin{array}{c} D_{1}^{\text{T}}D_{1}Q^{\text{T}}\hat{y}=\lambda D_{2}^{\text{T}}D_{2}Q^{\text{T}}\hat{y}. \end{array}$$

Letting $z=Q^{\text{T}}\hat{y}$, we need to solve

(Equation 11) $$\begin{array}{c} D_{1}^{\text{T}}D_{1}z=\lambda D_{2}^{\text{T}}D_{2}z, \end{array}$$

$D_{1}^{\text{T}}D_{1}$ and $D_{2}^{\text{T}}D_{2}$ are real, nonnegative diagonal matrices. Assume that $D_{1}^{\text{T}}D_{1}=diag\left(d_{11}^{2},\ldots ,d_{1r_{0}}^{2}\right)$ and $D_{2}^{\text{T}}D_{2}=diag\left(d_{21}^{2},\ldots ,d_{2r_{0}}^{2}\right)$. If the rank of B is $r_{0}$ $\left(l=r_{0}\right)$, all generalized singular values $d_{1i}/d_{2i}$ are finite; conversely, if $l<r_{0}$, the generalized singular values are infinite for $i=1,\ldots ,r_{0}-l$ and finite thereafter. When $d_{1i}/d_{2i}$ is finite $\left(d_{2i}\neq 0\right)$, the corresponding $z_{i}$ are the unit coordinate vectors. Thus, ${\hat{y}}_{i}={\left(Q^{\text{T}}\right)}^{-1}z_{i}$ and the new directions are given by

(Equation 12) $$\begin{array}{c} y_{i}=V_{r_{0}}{\left(Q^{\text{T}}\right)}^{-1}z_{i}, \end{array}$$

$y_{i}$ is the i-th column of $V_{r_{0}}{\left(Q^{\text{T}}\right)}^{-1}\;for\;i=r_{0}-l+1,\ldots ,r_{0}$. When $i=1,\ldots ,r_{0}-l$, $d_{1i}/d_{2i}$ is infinite $\left(d_{2i}=0\right)$, we similarly select $y_{i}$ using the i-th column of $V_{r_{0}}{\left(Q^{\text{T}}\right)}^{-1}$. These represent directions that are missing entirely from the $W_{0}H_{0}$ factorization.

Typically, GSVD-NMF has the capacity to suggest missing directions only for rank-2 NMF and higher. For rank-1 factorizations, NMF algorithms find the global optimum, and since the first component in the SVD of a nonnegative matrix is nonnegative, the rank-1 NMF solution should already be equivalent to the first SVD component.

After fixing Y, the corresponding additional components for $W_{0}$ (defined as S) are derived from a least-squares problem by minimizing

(Equation 13) $$\begin{array}{c} E_{1}\left(S,\alpha ;X,W_{0},H_{0}\right)=\|X-\overset{r_{0}}{\underset{p=1}{\sum }}\alpha_{p}w_{0p}h_{0p}^{\text{T}}-SY{\|}^{2}, \end{array}$$

where $E_{1}:R_{+}^{m\times k}\times R_{+}^{r_{0}}\to R_{+}$, $w_{0p}$ is the p-th column in $W_{0}$, $h_{0p}$ is the p-th row in $H_{0}$ and $\alpha =\left[\alpha_{1},\alpha_{2},\ldots ,\alpha_{r_{0}}\right]$. Here α is required to be nonnegative in order to preserve the nonnegativity of the original components $W_{0}H_{0}$. The vector α is introduced to modulate the magnitude of the pre-existing components, thereby improving the adaptability of the matrix S in the minimization of Equation 13. The joint optimization problem over S and α in Equation 13, can be formulated as a standard Non Negative Least Squares (NNLS) problem. By defining the variable vector θ as the concatenation of the column-flattened S and α, the minimization becomes

(Equation 14) $$\begin{array}{c} E_{1}\left(\theta ;X_{\text{v}},Y,M_{\text{v}}\right)=\|X_{\text{v}}-F\theta {\|}^{2}, \end{array}$$

where $X_{\text{v}}\in R^{mn}$ is the columnwise flattening of X, $F=\left[Y^{\text{T}}⊗I_{\text{m}},M_{\text{v}}\right]\in R^{mn\times \left(mk+r_{0}\right)}$, the coefficient vector $\theta ={\left[m^{\text{T}},\alpha^{\text{T}}\right]}^{\text{T}}\in R^{mk+r_{0}}$, where $m={\left[s_{1}^{\text{T}},s_{2}^{\text{T}},\ldots ,s_{k}^{\text{T}}\right]}^{\text{T}}$ is the column-flattened S. Prior to solving the NNLS problem, Y must be made nonnegative. We employ a truncation strategy where the nonnegative residual matrix is $\Delta X=max\left(X-W_{0}H_{0},0\right)$, and the truncated result for the p-th row in Y is

(Equation 15) $$\begin{array}{c} y_{p+}=\left\{\begin{array}{c} max\left(y_{p},0\right)\;if\|\Delta X\cdot max\left(y_{p},0\right)\|\geq \|\Delta X\cdot max\left(-y_{p},0\right)\|\\ max\left(-y_{p},0\right)\;else \end{array}\right), \end{array}$$

where ‖v‖ denotes the 1-norm of the vector v. This truncation maximizes the alignment (1-norm) of the resulting nonnegative component $y_{p+}$ with the nonnegative residual ΔX, thus ensuring the effective in minimizing the remaining error. This single NNLS problem achieves a global optimum for S and α given a fixed Y without any other step. However, the computational cost is significant, particularly for large datasets. The temporal complexity of forming F is $O\left(m^{2}nk\right)$, which can be equivalent to hundreds of iterations of NMF algorithms(HALS). Solving the resulting NNLS problem in Equation 14 incurs additional cost.

Given this substantial computational burden, especially when the dimensions m and n are large, we propose an alternative, decoupled approach to accelerate the initialization process in practice. Expanding Equation 13 as

(Equation 16) $$\begin{array}{cc} E_{1}\left(m,\alpha ;\Theta ,\xi ,\phi ,\gamma ,P,\Psi \right)=\|X-\overset{r_{0}}{\underset{p=1}{\sum }}\alpha_{p}w_{0p}h_{0p}^{\text{T}}-SY{\|}^{2}=\\ & \alpha^{\text{T}}\Theta \alpha -2\xi^{\text{T}}\alpha +\phi -2\gamma^{\text{T}}m+2\alpha^{\text{T}}Pm+m^{\text{T}}\Psi m, \end{array}$$

where $\Theta \in R_{+}^{r_{0}\times r_{0}}$ with $\Theta =\left(W_{0}^{\text{T}}W_{0}\right)⊙\left(H_{0}H_{0}^{\text{T}}\right)$. $\xi \in R_{+}^{r_{0}}$ with $\xi_{p}=w_{0p}^{\text{T}}Xh_{0p}$, $\phi =\sum_{i,j}X_{ij}^{2}$, $\gamma ={\left[{\left(Xy_{1}\right)}^{\text{T}},{\left(Xy_{2}\right)}^{\text{T}},\ldots ,{\left(Xy_{k}\right)}^{\text{T}}\right]}^{\text{T}}$. P and Ψ are a block matrices, where $P_{pp^{\prime }}=h_{0p}^{\text{T}}y_{p^{\prime }}w_{0p}^{\text{T}}$ and $\Psi_{pp^{\prime }}=y_{p}^{\text{T}}y_{p^{\prime }}I_{m}$, $I_{m}\in R_{+}^{m\times m}$ is an identity matrix. Details about obtaining Θ,ξ,ϕ,γ,P,Ψ and Equation 16 are presented in Supplementary Materials. The stationary point of Equation 16 with respect to m is

(Equation 17) $$\begin{array}{c} m=\Psi^{-1}\left(\gamma -P^{\text{T}}\alpha \right). \end{array}$$

Substituting Equation 17 into Equation 16 yields

(Equation 18) $$\begin{array}{c} {\hat{E}}_{1}\left(\alpha ;\Theta ,\xi ,\phi ,\gamma ,P,\Psi \right)=\alpha^{\text{T}}\left(\Theta -P\Psi^{-1}P^{\text{T}}\right)\alpha -2\left(\xi^{\text{T}}-\gamma^{\text{T}}\Psi^{-1}P^{\text{T}}\right)\alpha +\phi -\gamma^{\text{T}}\Psi^{-1}\gamma , \end{array}$$

where ${\hat{E}}_{1}:R_{+}^{r_{0}}\to R_{+}$. Since Equation 18 is derived from a NNLS problem 16, the quadratic in Equation 18 must be positive semidefinite, and thus this problem is convex and admits a deterministic solution.^54^^,^^55^

Unlike the standard SVD, where singular vectors form orthogonal bases, the new components derived through GSVD-based feature recovery are not guaranteed to be orthogonal. The suggested components are unique, up to scaling, if the generalized singular values are distinct. GSVD-NMF cannot make the solution worse:

**Lemma 1**. Given a data matrix X and an existing approximate factorization $W_{0}H_{0}$, the optimal solution $\alpha_{1}^{*},\ldots ,\alpha_{r_{0}}^{*},S^{*}$ to Equation 16 cannot increase the fitting error.

*Proof*. This follows from the fact that the solution $W_{0}H_{0}$ lies within the solution space:

(Equation 19) $$\begin{array}{cc} \|X-\overset{r_{0}}{\underset{p=1}{\sum }}\alpha_{p}^{*}w_{0p}h_{0p}^{\text{T}}-S^{*}Y{\|}^{2} & \leq \|X-\overset{r_{0}}{\underset{p=1}{\sum }}1\cdot w_{0p}h_{0p}^{\text{T}}-0Y{\|}^{2}\\ & =\|X-W_{0}H_{0}{\|}^{2}. \end{array}$$

We note that if the initial factorization is exact, then all generalized singular values are 1, Y is arbitrary, S will be zero, and the fitting error will not be decreased. Thus we cannot guarantee a decrease in error, but we can at least guarantee a non-increasing error.

To impose nonnegativity constraints on S and Y from Equation 17 and Equation 18 without significantly increasing the computational demand, we use the truncation step of NNDSVD, which generates the best nonnegative approximation of each new component.^34^ While ensuring the nonnegativity, in principle this comes with a risk: what if truncation results in an all-zero new component? In this circumstance, no useful work would be done. Here, we demonstrate that this cannot occur:

**Lemma 2**. *Given a data matrix* X *and an existing locally-optimal nonnegative factorization* $W_{0}H_{0}$ *for which* $X\neq W_{0}H_{0}$*, the nonnegative solution minimizing* Equation 16 *strictly reduces the error.*

*Proof*. The residual between the original matrix and the existing under-complete NMF solution, given by $R=X-W_{0}H_{0}$, contains both positive and negative values.^19^ Let i,j be a specific entry satisfying $R_{ij}>0$, and let $S=e_{i}$ and $Y=R_{ij}e_{j}^{T}$. This has positive components and reduces the sum-of-squares of the residual even in the sub-optimal case of α=1. Thus, there exists a nontrivial nonnegative component that will reduce the residual. Since the GSVD improves on this ansatz, and NNDSVD provides its optimal nonnegative rank-1 approximation, this procedure cannot return an all-zeros component unless the residual is itself zero.

Since the truncation alters the entries in S and Y, we let $W=\left[W_{0}diag\left(\alpha \right)\;W_{\text{new}}\right]$ and $H=\left[H_{0};\;H_{\text{new}}\right]$, all components in W and H are re-optimized together by minimizing

(Equation 20) $$E_{2}\left(\beta ;X,W,H\right)=\|X-\overset{r_{0}+k}{\underset{p=1}{\sum }}\beta_{p}w_{p}h_{p}^{\text{T}}{\|}^{2},$$

where $E_{2}:R_{+}^{r_{0}+k}\to R_{+}$, $\beta =\left[\beta_{1},\beta_{2},\ldots ,\beta_{r_{0}+k}\right]$. $w_{p}$, $h_{p}$ are the p-th column and p-th row in W, H respectively. Equation 20 is also a NNLS problem and thus convex. Moreover, this step also guarantees that the fitting error in Equation 20 does not exceed that of the under-complete NMF, following an analysis analogous to Equation 19. The final step is refinement via NMF, initialized with $W_{g}=Wdiag\left(\beta \right)$ and $H_{g}=H$. The GSVD-based feature recovery is summarized in Algorithm 1.

Algorithm 1 **GSVDFeatureRecovery**

**Input:** $W_{0},\;H_{0},\;U_{r_{0}},\;\Sigma_{r_{0}},\;V_{r_{0}},\;k$

**Output:** $W_{g},\;H_{g}$

 Compute Q, C, G, $D_{1}$, $D_{2}$ by solving Equation 8 and Equation 9

 
l←size(G,1)

 
$\lambda \leftarrow vcat\left(\mathrm{fi}ll\left(Inf,r_{0}-l\right),\;diag\left({\left(G^{\text{T}}G\right)}^{-1}C^{\text{T}}C\right)\right)$

 Y←k columns in $V_{r_{0}}{\left(Q^{\text{T}}\right)}^{-1}$ corresponding to the largest k values in λ

 Compute S,α with Equation 17 and Equation 18

 Compute $W_{\text{new}}$, $H_{\text{new}}$ by truncating S, Y (Truncation step in NNDSVD)

 
$W\leftarrow \left[W_{0}diag\left(\alpha \right)\;W_{\text{new}}\right]$

 
$H\leftarrow \left[H_{0};\;H_{\text{new}}\right]$

 Compute β with Equation 20

 
$W_{g}\leftarrow Wdiag\left(\beta \right)$

 
$H_{g}\leftarrow H$

If the SVD in Equation 6 has more components than the NMF, it is guaranteed that some singular values λ will be infinite, and our strategy will first select directions missing from the NMF. When adopting this strategy, the schematic in Figure 1 depicting the polishing of existing directions is no longer relevant.

#### Temporal complexity analysis

The GSVD-based feature recovery is a one-shot procedure that requires no iterative optimization. To analyze its temporal complexity, we divide it into three main components.

For initializing new columns in H (denoted as “init H” in Figure S1), the dominant computational terms arise from (i) computing $BVr_{0}$ in Equation 7, (ii) performing the GSVD in Equation 8, and (iii) evaluating $Vr_{0}{\left(Q^{\text{T}}\right)}^{-1}$ in Equation 12. The temporal complexities of these steps are $O\left(\left(m+n\right)r_{0}^{2}\right)$, $O\left(\left(m+r_{0}\right)r_{0}^{2}+r_{0}^{3}\right)$, and $O\left(r_{0}^{3}+nr_{0}^{2}\right)$, respectively. Consequently, the overall complexity of this step can be summarized as $O\left(m+n+r_{0}\right)r_{0}^{2}$. Given that $r_{0}\ll \min\left(m,n\right)$, the dominant complexity simplifies to approximately $O\left(\left(m+n\right)r_{0}^{2}\right)$.

For initializing new rows in W (“init W” in Figure S1), the major computations include evaluating the coefficients $\Theta -P\Psi^{-1}P^{\text{T}}$ and $\xi^{\text{T}}-\gamma^{\text{T}}\Psi^{-1}P^{\text{T}}$ in Equation 18, solving the nonnegative least-squares problem in the same equation, and forming new rows according to Equation 17. The coefficient computation in Equation 18 dominates, with a temporal complexity of $O\left(mn\left(r_{0}+k\right)+\left(m+n\right)r_{0}^{2}\right)$ as detailed in the Supplementary Materials. Minimizing Equation 18 requires $O\left(tr_{0}^{2}\right)$ time, where t denotes the number of iterations—typically on the order of tens—thus negligible compared to the coefficient computation. Constructing new rows via Equation 17 costs $O\left(\left(mnk+\left(m+n\right)r_{0}k\right)\right)$. Therefore, the overall dominant temporal complexity of “init W” is O(mnr).

For the amplitude multiplication step, which is analogous to solving the nonnegative least-squares problem for initializing new rows in W, the temporal complexity is $O\left(t{\left(r_{0}+k\right)}^{2}\right)$, where t is the number of iterations (typically in the order of dozens).

In summary, the overall dominant temporal complexity of the GSVD-based feature recovery is O(mnr), which is comparable to the complexity of a single iteration of the HALS algorithm for NMF.^17^ Empirically, the total runtime of the GSVD-based feature recovery corresponds to roughly two to three iterations of the NMF algorithm. Therefore, this method is significantly more efficient than restarting NMF from scratch. Moreover, as shown in later experiments, since the NMF algorithm converges rapidly when initialized with our GSVD-based recovery, expanding the rank of NMF using our approach is substantially more efficient than reinitializing NMF entirely.

#### The whole pipeline

The whole pipeline of the proposed method is given in Figure S1 in Supplementary Materials. Given that standard NMF typically yields local optima and SVD constitutes a global optimum of matrix factorization, our core concept involves identifying features captured by SVD but absent in an existing approximate factorization. These identified features are then incorporated as new components into the existing NMF results. $W_{0}$ and $H_{0}$ are the results of under-complete NMF with $r_{0}$ components. We use $U_{r_{0}}$, $\Sigma_{r_{0}}$ and $V_{r_{0}}$ to denote the results of rank-$r_{0}$ approximate SVD, which is the global optimum of rank-$r_{0}$ matrix factorization of X. Using the GSVD, we compare the results of under-complete NMF and rank-$r_{0}$ SVD, and on that basis propose new rows (denoted Y) for H and new columns (denoted S) for W. The matrices S and Y are not guaranteed to be nonnegative, so NNDSVD truncation^34^ is applied to preserve the nonnegative approximation. Additionally, the amplitudes of the original and newly added components are jointly adjusted using a nonnegative least-squares approach to balance the power between the existing and new components. Finally, all components are polished by NMF to produce the final factorization. The pseudo-code for the full GSVD-NMF pipeline is provided in Algorithm 2. We use the notation “NMF(X,r,ϵ,W,H)” to denote running NMF on matrix X with r components and convergence relative tolerance ϵ, initialized with W and H. The procedure “GSVDFeatureRecovery(·)”, detailed in the next subsection and in Algorithm 1, performs the GSVD-based component augmentation. If an under-complete NMF solution ($W_{0}$, $H_{0}$) is already available, rank expansion can be performed directly by invoking “GSVDFeatureRecovery(·)”.

Algorithm 2 **GSVD-NMF**

**Input:** $X,W_{00},H_{00},r_{0},k$;

**Output:** $W_{1},H_{1}$

 
$W_{0},H_{0}\leftarrow NMF\left(X,r_{0},\epsilon_{0},W_{00},H_{00}\right)$

 
$U_{r_{0}},\Sigma_{r_{0}},V_{r_{0}}\leftarrow SVD\left(X,r_{0}\right)$

 
$W_{\text{g}},H_{\text{g}}\leftarrow GSVDFeatureRecovery\left(W_{0},H_{0},k,U_{r_{0}},\Sigma_{r_{0}},V_{r_{0}}\right)$

 
$W_{1},H_{1}\leftarrow NMF\left(X,r_{0}+k,\epsilon_{1},W_{\text{init}},H_{\text{init}}\right)$

It can be seen that a truncation step occurs in the GSVD-based feature-recovery stage. However, the Multiplicative Update (MU) algorithm is known to be sensitive to zero entries and scaling effects, which can prevent the newly introduced components from being properly activated and normalized.^56^ To mitigate this issue, when using MU or MU-related NMF algorithms, we add a small perturbation $\left(10^{-5}\right)$ to all components before the final NMF refinement.

### Quantification and statistical analysis

On real-world data, in addition to HALS we also analyzed Greedy Coordinate Descent (GCD),^57^ Alternating Least Squares Using Projected Gradient Descent (ALSPGrad),^18^ and Multiplicative Updating (MU).^58^ After establishing efficacy with a variety of algorithms, later analysis on computational efficiency and influence of parameters is performed just with HALS. To determine convergence, here we monitor the maximum relative change in the Frobenius norm of the columns of $W\in R_{+}^{m\times r}$ and the rows of $H\in R_{+}^{r\times n}$ (given in Equation 21), and stop iterating when

(Equation 21) $$\begin{array}{cc} \|w_{j}^{\left(k+1\right)}-w_{j}^{\left(k\right)}{\|}_{\text{F}}^{2} & \leq \epsilon \|w_{j}^{\left(k+1\right)}+w_{j}^{\left(k\right)}{\|}_{\text{F}}^{2},\\ \|h_{j}^{\left(k+1\right)}-h_{j}^{\left(k\right)}{\|}_{\text{F}}^{2} & \leq \epsilon \|h_{j}^{\left(k+1\right)}+h_{j}^{\left(k\right)}{\|}_{\text{F}}^{2}, \end{array}$$

for j=1,2,…,r, for some choice of ϵ. In these expressions, $w_{j}^{\left(k\right)}$ and $h_{j}^{\left(k\right)}$ are the j-th column and j-th row in W and H on the k-th iteration, respectively. Unless otherwise specified, $\epsilon =10^{-4}$ was used. Throughout, the maximum number of iterations for NMF was set so high that termination was triggered only by the ϵ-criterion. The relative fitting error between WH and the input matrix X, $100\|X-WH{\|}_{2}^{2}/\|X{\|}_{2}^{2}$, was used to evaluate the local optima of NMF. This measures how well the factorization fit the input matrix.

Since the ground truth components of real-world data are undetermined, we assessed methods in terms of the value of the objective Equation 2, with smaller values indicating better solutions. For each random initialization, we compared the final solution from GSVD-NMF against standard NMF with the same number of components. Standard NMF was initialized with a random rank-r solution, with r given in Table 1. The under-complete NMF was initialized with the first $r_{0}=r-k$ components of this same initialization, and then k new components were added by GSVD-NMF to achieve rank r. Thus, the two solutions start from as much of a shared initialization as can be achieved given their differences in initial rank. This comparison addresses whether it is more effective and efficient to directly augment components for an existing under-complete NMF solution or to rerun NMF from scratch with additional components. Solution objective values are compared in scatter plots, where each dot represents a single random initialization, and brown lines represent the histogram of perpendicular distances between points and the diagonal (a.k.a., equal performance) in the figure. The total number of random initializations in this experiment is 200.

In addition to comparing the GSVD-NMF pipeline with standard NMF algorithms, we also evaluated it against other NMF methods that incrementally increase the rank, including Recursive NMU (RNMU),^59^ Global NMU (LNMU),^19^ Prior NMU (PNMU)^32^ and NMU-ADMM.^31^

The simulation and experiments were performed using Julia 1.10 on Washington University in St. Louis RIS scientific computing platform with Intel_Xeon_Gold6242CPU280GHz 8G RAM.
