# Supplementary material for: Recovering missing features in nonnegative matrix factorization via generalized singular value decomposition
Source: iScience. 2026 Feb 12;29(3):114708. doi: 10.1016/j.isci.2026.114708 (PMC12989961; doi:10.1016/j.isci.2026.114708)
Supplement: Document S1. Figures S1–S5 and Table S1 [file mmc1.pdf]

iScience, Volume *29*

Supplemental information

Recovering missing features in nonnegative matrix factorization via generalized singular value decomposition

Youdong Guo and Timothy E. Holy

SUPPLEMENTAL INFORMATION

The whole-pipeline figure

The figure of the whole-pipeline is shown as Figure S1.

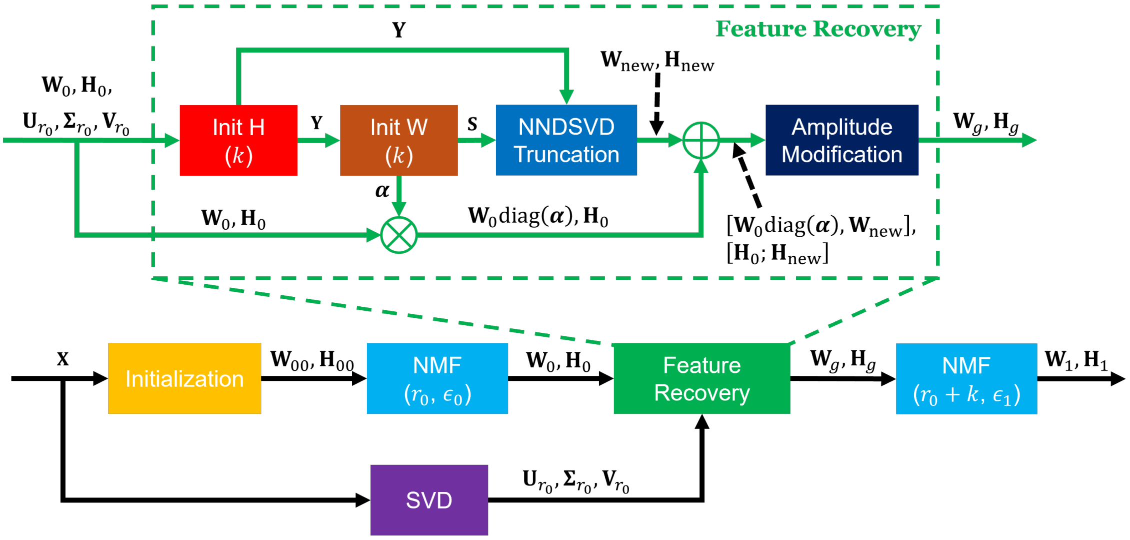

Figure S1: The whole pipeline of GSVD-NMF.

Derivation of the Equation 16 and Equation 18

In this section, we provide details about the derivations of Equation 16 and Equation 18. Supposing $\mathbf{H}$ is augmented with $\mathbf{Y} = [\mathbf{y}_1, \mathbf{y}_2, \cdots, \mathbf{y}_k]$ and $\mathbf{W}$ is augmented with $\mathbf{S} = [\mathbf{s}_1, \mathbf{s}_2, \cdots, \mathbf{s}_k]$, where $\mathbf{S}$ is obtained by minimizing Equation 16. Equation 16 can be rewritten as

$$
\begin{aligned}
&E_{sy}\left(\mathbf{S}, \mathbf{Y}, \boldsymbol{\alpha}; \mathbf{X}, \mathbf{W}_0, \mathbf{H}_0\right) \\
&\quad = \|\mathbf{X} - \sum_{p=1}^{r_0} \alpha_p \mathbf{M}_p - \sum_{z=1}^{k} \mathbf{s}_z \mathbf{y}_z^{\mathrm{T}}\|^2 \\
&\quad = \sum_{i=1}^{m}\sum_{j=1}^{n}\left[X_{ij} - \sum_{p=1}^{r_0}\alpha_p M_{p,ij}\right]^2 - 2\sum_{i=1}^{m}\sum_{j=1}^{n}\left[\left(X_{ij} - \sum_{p=1}^{r}\alpha_p M_{p,ij}\right)\left(\sum_{z=1}^{k} s_{z,i} y_{z,j}\right)\right] \\
&\quad + \sum_{i=1}^{m}\sum_{j=1}^{n}\left(\sum_{z=1}^{k} s_{z,i} y_{z,j}\right)^2,
\end{aligned} \tag{S1}
$$

where $\mathbf{X} \in \mathbb{R}_{+}^{m \times n}$, $\mathbf{M}_p = \mathbf{w}_{0p}\mathbf{h}_{0p}^{\mathrm{T}}$. $\mathbf{w}_{0p}$ and $\mathbf{h}_{0p}$ denote the p-th column and row in $\mathbf{W}_0$ and $\mathbf{H}_0$, respectively. The first term of Equation S1 can be written as

$$\begin{aligned}
&\sum_{i=1}^{m}\sum_{j=1}^{n}\left[X_{ij} - \sum_{p=1}^{r_0}\alpha_p M_{p,ij}\right]^2 \\
&= \sum_{i=1}^{m}\sum_{j=1}^{n}\left(X_{ij}\right)^2 - 2\sum_{p=1}^{r_0}\alpha_p\sum_{i=1}^{m}\sum_{j=1}^{n}M_{p,ij}X_{ij} + \sum_{i=1}^{m}\sum_{j=1}^{n}\left(\sum_{p=1}^{r_0}\alpha_p M_{p,ij}\right)^2 \\
&= \phi - 2\boldsymbol{\xi}^{\mathrm{T}}\boldsymbol{\alpha} + \sum_{p=1}^{r_0}\sum_{p'=1}^{r_0}\alpha_p\left(\sum_{i=1}^{m}\sum_{j=1}^{n}M_{p,ij}M_{p',ij}\right)\alpha_{p'} \\
&= \boldsymbol{\alpha}^{\mathrm{T}}\boldsymbol{\Theta}\boldsymbol{\alpha} - 2\boldsymbol{\xi}^{\mathrm{T}}\boldsymbol{\alpha} + \phi,
\end{aligned} \tag{S2}$$

where $\boldsymbol{\alpha} \in \mathbb{R}_{+}^{r_0}$, $\phi = \sum_{i,j} X_{ij}^2$, $\boldsymbol{\xi} \in \mathbb{R}_{+}^{r_0}$ and $\xi_p = \mathbf{w}_{0p}^{\mathrm{T}}\mathbf{X}\mathbf{h}_{0p}$, $\boldsymbol{\Theta} \in \mathbb{R}_{+}^{r_0 \times r_0}$ and $\boldsymbol{\Theta} = \left(\mathbf{W}_0^{\mathrm{T}}\mathbf{W}_0\right) \odot \left(\mathbf{H}_0\mathbf{H}_0^{\mathrm{T}}\right)$. The second term in Equation S1 can be written as

$$\begin{aligned}
&\sum_{i=1}^{m}\sum_{j=1}^{n}\left[\left(X_{ij} - \sum_{p=1}^{r}\alpha_p M_{p,ij}\right)\left(\sum_{z=1}^{k}s_{z,i}y_{z,j}\right)\right] \\
&= \sum_{i=1}^{m}\sum_{j=1}^{n}X_{ij}\sum_{z=1}^{k}s_{z,i}y_{z,j} - \sum_{i=1}^{m}\sum_{j=1}^{n}\left[\left(\sum_{p=1}^{r_0}\alpha_p M_{p,ij}\right)\left(\sum_{z=1}^{k}s_{z,i}y_{z,j}\right)\right] \\
&= \sum_{z=1}^{k}\mathbf{s}_z^{\mathrm{T}}\mathbf{X}\mathbf{y}_z - \sum_{p=1}^{r_0}\alpha_p\sum_{z=1}^{k}\left(\mathbf{s}_z^{\mathrm{T}}\mathbf{w}_{0p}\right)\left(\mathbf{h}_{0p}^{\mathrm{T}}\mathbf{y}_z\right).
\end{aligned} \tag{S3}$$

The third term in Equation S1 can be written as

$$\begin{aligned}
\sum_{i=1}^{m}\sum_{j=1}^{n}\left(\sum_{z=1}^{k}s_{z,i}y_{z,j}\right)^2 &= \sum_{i=1}^{m}\sum_{j=1}^{n}\left(\sum_{z=1}^{k}s_{z,i}y_{z,j}\right)\left(\sum_{k'=1}^{k}s_{k',i}y_{k',j}\right) \\
&= \sum_{z=1}^{k}\sum_{k'=1}^{k}\left(\sum_{i=1}^{m}s_{z,i}s_{k',i}\sum_{j=1}^{n}y_{z,j}y_{k',j}\right) \\
&= \sum_{z=1}^{k}\sum_{k'=1}^{k}\mathbf{s}_z^{\mathrm{T}}Y_{zk'}^{\#}\mathbf{s}_{k'},
\end{aligned} \tag{S4}$$

where $\mathbf{Y}^{\#} \in \mathbb{R}^{k \times k}$ and $Y_{zk'}^{\#} = \mathbf{y}_z^{\mathrm{T}}\mathbf{y}_{k'}$. Therefore, Equation S1 can be rewritten as

$$\begin{aligned}
&E_{sy}\left(\mathbf{S}, \mathbf{Y}, \boldsymbol{\alpha}; \mathbf{X}, \mathbf{W}_0, \mathbf{H}_0\right) \\
&= \boldsymbol{\alpha}^{\mathrm{T}}\boldsymbol{\Theta}\boldsymbol{\alpha} - 2\boldsymbol{\xi}^{\mathrm{T}}\boldsymbol{\alpha} + \phi - 2\sum_{z=1}^{k}\mathbf{s}_z^{\mathrm{T}}\mathbf{X}\mathbf{y}_z + 2\sum_{p=1}^{r_0}\alpha_p\sum_{z=1}^{k}\left(\mathbf{s}_z^{\mathrm{T}}\mathbf{w}_{0p}\right)\left(\mathbf{h}_{0p}^{\mathrm{T}}\mathbf{y}_z\right) + \sum_{z=1}^{k}\sum_{k'=1}^{k}\mathbf{s}_z^{\mathrm{T}}Y_{zk'}^{\#}\mathbf{s}_{k'}.
\end{aligned} \tag{S5}$$

To minimize Equation S5, we first vectorize $\mathbf{S}$ by letting $\mathbf{m} = \left[\mathbf{s}_1^{\mathrm{T}}, \mathbf{s}_2^{\mathrm{T}}, \ldots, \mathbf{s}_k^{\mathrm{T}}\right]^{\mathrm{T}}$, where $\mathbf{m} \in \mathbb{R}_{+}^{mk}$. Then we have

$$\begin{aligned}
\sum_{z=1}^{k}\mathbf{s}_z^{\mathrm{T}}\mathbf{X}\mathbf{y}_z &= \left[\mathbf{s}_1^{\mathrm{T}}, \mathbf{s}_2^{\mathrm{T}}, \ldots, \mathbf{s}_k^{\mathrm{T}}\right]\left[\left(\mathbf{X}\mathbf{y}_1\right)^{\mathrm{T}}, \left(\mathbf{X}\mathbf{y}_2\right)^{\mathrm{T}}, \ldots, \left(\mathbf{X}\mathbf{y}_k\right)^{\mathrm{T}}\right]^{\mathrm{T}} \\
&= \mathbf{m}^{\mathrm{T}}\boldsymbol{\gamma},
\end{aligned} \tag{S6}$$

where $\gamma = \left[(\mathbf{X}\mathbf{y}_1)^{\mathrm{T}}, (\mathbf{X}\mathbf{y}_2)^{\mathrm{T}}, \ldots, (\mathbf{X}\mathbf{y}_k)^{\mathrm{T}}\right]^{\mathrm{T}}$ and $\gamma \in \mathbb{R}_+^{mk}$. The fifth term of Equation S5 is

$$\begin{aligned}\sum_{p=1}^{r_0} \alpha_p \sum_{z=1}^{k} \left(\mathbf{s}_z^{\mathrm{T}} \mathbf{w}_{0p}\right) \left(\mathbf{h}_{0p}^{\mathrm{T}} \mathbf{y}_z\right) &= \sum_{p=1}^{r} \sum_{z=1}^{k} \alpha_p \left(\mathbf{h}_{0p}^{\mathrm{T}} \mathbf{y}_z \mathbf{w}_{0p}^{\mathrm{T}}\right) \mathbf{s}_z \\ &= \boldsymbol{\alpha}^{\mathrm{T}} \mathbf{P}\mathbf{m},\end{aligned} \tag{S7}$$

where

$$\mathbf{P} = \begin{bmatrix} \mathbf{h}_{01}^{\mathrm{T}}\mathbf{y}_1\mathbf{w}_{01}^{\mathrm{T}} & \mathbf{h}_{01}^{\mathrm{T}}\mathbf{y}_2\mathbf{w}_{01}^{\mathrm{T}} & \ldots & \mathbf{h}_{01}^{\mathrm{T}}\mathbf{y}_k\mathbf{w}_{01}^{\mathrm{T}} \\ \mathbf{h}_{02}^{\mathrm{T}}\mathbf{y}_1\mathbf{w}_{02}^{\mathrm{T}} & \mathbf{h}_{02}^{\mathrm{T}}\mathbf{y}_2\mathbf{w}_{02}^{\mathrm{T}} & \ldots & \mathbf{H}_{2}^{\mathrm{T}}\mathbf{y}_k\mathbf{w}_{2}^{\mathrm{T}} \\ \vdots & \vdots & \ddots & \vdots \\ \mathbf{h}_{0r}^{\mathrm{T}}\mathbf{y}_1\mathbf{w}_{0r}^{\mathrm{T}} & \mathbf{h}_{0r}^{\mathrm{T}}\mathbf{y}_2\mathbf{w}_{0r}^{\mathrm{T}} & \ldots & \mathbf{h}_{0r}^{\mathrm{T}}\mathbf{y}_k\mathbf{w}_{0r}^{\mathrm{T}} \end{bmatrix}_{r_0 \times mk}. \tag{S8}$$

The last term of Equation S5 is

$$\sum_{z=1}^{k} \sum_{k'=1}^{k} \mathbf{s}_z^{\mathrm{T}} Y_{zk'}^{\#} \mathbf{s}_{k'} = \mathbf{m}^{\mathrm{T}} \boldsymbol{\Psi} \mathbf{m}, \tag{S9}$$

where $\boldsymbol{\Psi} = \mathbf{Y}^{\#} \otimes \mathbf{I}_m$ and $\mathbf{I}_m \in \mathbb{R}_+^{m \times m}$ is identity matrix. Therefore,

$$\begin{aligned} E_{sy}\left(\mathbf{S}, \mathbf{Y}, \boldsymbol{\alpha}; \mathbf{X}, \mathbf{W}_0, \mathbf{H}_0\right) =& \boldsymbol{\alpha}^{\mathrm{T}} \boldsymbol{\Theta} \boldsymbol{\alpha} - 2\boldsymbol{\xi}^{\mathrm{T}} \boldsymbol{\alpha} + \phi - 2\mathbf{m}^{\mathrm{T}} \boldsymbol{\gamma} + 2\boldsymbol{\alpha}^{\mathrm{T}} \mathbf{P}\mathbf{m} + \mathbf{m}^{\mathrm{T}} \boldsymbol{\Psi} \mathbf{m} \\ =& E_1\left(\mathbf{m}, \boldsymbol{\alpha}; \boldsymbol{\Theta}, \boldsymbol{\xi}, \phi, \boldsymbol{\gamma}, \mathbf{P}, \boldsymbol{\Psi}\right). \end{aligned} \tag{S10}$$

Here, $E_1\left(\mathbf{m}, \boldsymbol{\alpha}; \boldsymbol{\Theta}, \boldsymbol{\xi}, \phi, \boldsymbol{\gamma}, \mathbf{P}, \boldsymbol{\Psi}\right)$ is Equation 16. Setting the partial derivative of Equation S10 with respect to $\mathbf{m}$ to zero yields

$$\frac{\partial E_1}{\partial \mathbf{m}} = -2\boldsymbol{\gamma}^{\mathrm{T}} + 2\boldsymbol{\alpha}^{\mathrm{T}} \mathbf{P} + 2\mathbf{m}^{\mathrm{T}} \boldsymbol{\Psi} = 0. \tag{S11}$$

Thus,

$$\mathbf{m} = \boldsymbol{\Psi}^{-1} \left(\boldsymbol{\gamma} - \mathbf{P}^{\mathrm{T}} \boldsymbol{\alpha}\right). \tag{S12}$$

Plugging Equation S12 back into Equation S10 yields

$$\begin{aligned} &\hat{E}_1\left(\boldsymbol{\alpha}; \boldsymbol{\Theta}, \boldsymbol{\xi}, \phi, \boldsymbol{\gamma}, \mathbf{P}, \boldsymbol{\Psi}\right) \\ &= \boldsymbol{\alpha}^{\mathrm{T}} \boldsymbol{\Theta} \boldsymbol{\alpha} - 2\boldsymbol{\xi}^{\mathrm{T}} \boldsymbol{\alpha} + \phi - \mathbf{m}^{\mathrm{T}} \boldsymbol{\Psi} \mathbf{m} \\ &= \boldsymbol{\alpha}^{\mathrm{T}} \boldsymbol{\Theta} \boldsymbol{\alpha} - 2\boldsymbol{\xi}^{\mathrm{T}} \boldsymbol{\alpha} + \phi - \boldsymbol{\gamma}^{\mathrm{T}} \boldsymbol{\Psi}^{-1} \boldsymbol{\gamma} + \boldsymbol{\gamma}^{\mathrm{T}} \boldsymbol{\Psi}^{-1} \mathbf{P}^{\mathrm{T}} \boldsymbol{\alpha} + \boldsymbol{\alpha}^{\mathrm{T}} \mathbf{P} \boldsymbol{\Psi}^{-1} \boldsymbol{\gamma} - \boldsymbol{\alpha}^{\mathrm{T}} \mathbf{P} \boldsymbol{\Psi}^{-1} \mathbf{P}^{\mathrm{T}} \boldsymbol{\alpha} \\ &= \boldsymbol{\alpha}^{\mathrm{T}} \left(\boldsymbol{\Theta} - \mathbf{P} \boldsymbol{\Psi}^{-1} \mathbf{P}^{\mathrm{T}}\right) \boldsymbol{\alpha} - 2\left(\boldsymbol{\xi}^{\mathrm{T}} - \boldsymbol{\gamma}^{\mathrm{T}} \boldsymbol{\Psi}^{-1} \mathbf{P}^{\mathrm{T}}\right) \boldsymbol{\alpha} + \phi - \boldsymbol{\gamma}^{\mathrm{T}} \boldsymbol{\Psi}^{-1} \boldsymbol{\gamma}. \end{aligned} \tag{S13}$$

Practical implementation

Although minimizing Equation S13 is a convex problem with a deterministic solution, directly computing each coefficient is computationally expensive. For example, given $\boldsymbol{\Theta}, \mathbf{P}$ and Ψ, directly computing the coefficient matrix $\boldsymbol{\Theta} - \mathbf{P}\boldsymbol{\Psi}^{-1}\mathbf{P}^{\mathrm{T}}$ in Equation S13 needs time of $O(m^2k^2r_0)$, a prohibitive cost. However, the block-diagonal structure of Ψ admits significant enhancements. For convenience, we first revisit the matrix dimensions: $\mathbf{X} \in \mathbb{R}^{m \times n}, \mathbf{W}_0 \in \mathbb{R}^{m \times r_0}, \mathbf{H}_0 \in \mathbb{R}^{r_0 \times n}, \mathbf{Y} \in \mathbb{R}^{k \times n}, \mathbf{Y}^{\#} \in \mathbb{R}^{k \times k}$. Letting $\bar{\mathbf{Y}}^{\#} = (\mathbf{Y}^{\#})^{-1}$, we have $\boldsymbol{\Psi}^{-1} = (\mathbf{Y}^{\#})^{-1} \otimes \mathbf{I}_{\mathbf{m}}$ and

$$\boldsymbol{\Psi}^{-1} = \begin{bmatrix} \bar{Y}_{11}^{\#}\mathbf{I}_m & \bar{Y}_{12}^{\#}\mathbf{I}_m & \ldots & \bar{Y}_{1k}^{\#}\mathbf{I}_m \\ \bar{Y}_{21}^{\#}\mathbf{I}_m & \bar{Y}_{22}^{\#}\mathbf{I}_m & \ldots & \bar{Y}_{2k}^{\#}\mathbf{I}_m \\ \vdots & \vdots & \ddots & \vdots \\ \bar{Y}_{k1}^{\#}\mathbf{I}_m & \bar{Y}_{k2}^{\#}\mathbf{I}_m & \ldots & \bar{Y}_{kk}^{\#}\mathbf{I}_m \end{bmatrix}_{mk \times mk}. \tag{S14}$$

Then,

$$\begin{aligned}\left(\mathbf{P}\boldsymbol{\Psi}^{-1}\mathbf{P}^{\mathrm{T}}\right)_{ij} &= \sum_{zz'} P_{iz}\bar{Y}^{\#}_{zz'}\mathbf{I}_m P_{jz'} \\ &= \sum_{zz'} \mathbf{h}_{0i}^{\mathrm{T}}\mathbf{y}_z\mathbf{w}_{0i}^{\mathrm{T}}\bar{Y}^{\#}_{zz'}\mathbf{w}_{0j}\mathbf{y}_{z'}^{\mathrm{T}}\mathbf{h}_{0j} \\ &= \mathbf{w}_{0i}^{\mathrm{T}}\mathbf{w}_{0j}\sum_{zz'} \mathbf{h}_{0i}^{\mathrm{T}}\mathbf{y}_z\bar{Y}^{\#}_{zz'}\mathbf{y}_{z'}^{\mathrm{T}}\mathbf{h}_{0j} \\ &= \mathbf{w}_{0i}^{\mathrm{T}}\mathbf{w}_{0j}\left(\left(\mathbf{H}_0\mathbf{Y}^{\mathrm{T}}\right)\bar{\mathbf{Y}}^{\#}\left(\mathbf{H}_0\mathbf{Y}^{\mathrm{T}}\right)^{\mathrm{T}}\right)_{ij}.\end{aligned} \tag{S15}$$

Thus,

$$\mathbf{P}\boldsymbol{\Psi}^{-1}\mathbf{P}^{\mathrm{T}} = \left(\mathbf{W}_0^{\mathrm{T}}\mathbf{W}_0\right)\odot\left(\left(\mathbf{H}_0\mathbf{Y}^{\mathrm{T}}\right)\bar{\mathbf{Y}}^{\#}\left(\mathbf{H}_0\mathbf{Y}^{\mathrm{T}}\right)^{\mathrm{T}}\right). \tag{S16}$$

Therefore,

$$\boldsymbol{\Theta} - \mathbf{P}\boldsymbol{\Psi}^{-1}\mathbf{P}^{\mathrm{T}} = \left(\mathbf{W}_0^{\mathrm{T}}\mathbf{W}_0\right)\odot\left(\mathbf{H}_0\mathbf{H}_0^{\mathrm{T}} - \left(\mathbf{H}_0\mathbf{Y}^{\mathrm{T}}\right)\bar{\mathbf{Y}}^{\#}\left(\mathbf{H}_0\mathbf{Y}^{\mathrm{T}}\right)^{\mathrm{T}}\right). \tag{S17}$$

The temporal complexity of Equation S17 is dominated by matrix multiplications, yielding $O((m+n)r_0^2 + nr_0k + r_0^2k + r_0k^2)$. Since, $r_0 \ll \min(m,n)$ and $k < r_0$ (sometimes $k \ll r_0$), the dominant term simplifies to $O((m+n)r_0^2)$.

For the second term in Equation S13, since $\xi_p = \mathbf{w}_{0p}^{\mathrm{T}}\mathbf{X}\mathbf{h}_{0p}$, we have $\boldsymbol{\xi} = \operatorname{diag}\left(\mathbf{W}_0^{\mathrm{T}}\mathbf{X}\mathbf{H}_0^{\mathrm{T}}\right)$. Then,

$$\begin{aligned}\left(\boldsymbol{\gamma}^{\mathrm{T}}\boldsymbol{\Psi}^{-1}\mathbf{P}^{\mathrm{T}}\right)_p &= \sum_{zz'}(\mathbf{X}\mathbf{y}_k)^{\mathrm{T}}\bar{Y}^{\#}_{zz'}\mathbf{w}_{0p}\mathbf{y}_{z'}^{\mathrm{T}}\mathbf{h}_{0p} \\ &= \sum_{zz'}\mathbf{h}_{0p}^{\mathrm{T}}\mathbf{y}_z\bar{Y}^{\#}_{zz'}\mathbf{w}_{0p}^{\mathrm{T}}\mathbf{X}\mathbf{y}_k \\ &= \left(\mathbf{H}_0\mathbf{Y}^{\mathrm{T}}\right)_{pz}\bar{Y}^{\#}_{zz'}\left(\mathbf{Y}\mathbf{X}^{\mathrm{T}}\mathbf{W}_0\right)_{z'p}.\end{aligned} \tag{S18}$$

Thus, $\boldsymbol{\gamma}^{\mathrm{T}}\boldsymbol{\Psi}^{-1}\mathbf{P}^{\mathrm{T}} = \operatorname{diag}\left(\left(\mathbf{H}_0\mathbf{Y}^{\mathrm{T}}\right)\bar{\mathbf{Y}}^{\#}\left(\mathbf{Y}\mathbf{X}^{\mathrm{T}}\mathbf{W}_0\right)\right)$, where $\operatorname{diag}(\cdot)$ denotes collecting the diagonal elements in the matrix. And therefore,

$$\boldsymbol{\xi}^{\mathrm{T}} - \boldsymbol{\gamma}^{\mathrm{T}}\boldsymbol{\Psi}^{-1}\mathbf{P}^{\mathrm{T}} = \operatorname{diag}\left(\mathbf{W}_0^{\mathrm{T}}\mathbf{X}\mathbf{H}_0^{\mathrm{T}} - \left(\mathbf{H}_0\mathbf{Y}^{\mathrm{T}}\right)\bar{\mathbf{Y}}^{\#}\left(\mathbf{Y}\mathbf{X}^{\mathrm{T}}\mathbf{W}_0\right)\right). \tag{S19}$$

The temporal complexity of Equation S19 is again dominated by matrix multiplications, giving $O(mnr_0 + nr_0^2 + nr_0k + r_0k^2 + mnk + mkr_0 + r_0^2k)$. Similar to the analysis of the first term, the dominant temporal complexity reduces to $O(mn(r_0+k))$.

The third term in Equation S13 is constant and does not involve $\boldsymbol{\alpha}$; thus, it does not affect the overall temporal complexity. In conclusion, the overall temporal complexity is dominated by $O(mn(r_0+k) + (m+n)r_0^2)$.

After $\boldsymbol{\alpha}$ is obtained, $\mathbf{S}$ is formed by reshaping $\mathbf{m}$ from Equation S12 to a $m \times k$ matrix. From the Equation S12, we have

$$\begin{aligned}\mathbf{s}_p &= \sum_z \bar{Y}^{\#}_{pz}\mathbf{X}\mathbf{y}_z - \sum_{zz'}\bar{Y}^{\#}_{pz}\mathbf{w}_{0z'}\mathbf{y}_z^{\mathrm{T}}\mathbf{h}_{0z'}\alpha_{z'} \\ &= \sum_z \mathbf{X}\mathbf{y}_z\bar{Y}^{\#}_{zp} - \sum_{zz'}\mathbf{w}_{0z'}\alpha_{z'}\mathbf{h}_{0z'}^{\mathrm{T}}\mathbf{y}_z\bar{Y}^{\#}_{zp} \\ &= \sum_z \left[\mathbf{X}\mathbf{Y}^{\mathrm{T}}\right]_{:,z}\bar{Y}^{\#}_{zp} - \sum_{zz'}\left[\mathbf{W}_0\mathrm{Diagonal}(\boldsymbol{\alpha})\right]_{:,z'}\left[\mathbf{H}_0\mathbf{Y}^{\mathrm{T}}\right]_{z'z}\bar{Y}^{\#}_{zp},\end{aligned} \tag{S20}$$

where $\mathrm{Diagonal}(\boldsymbol{\alpha}) = \begin{bmatrix} \alpha_1 & 0 & \dots & 0 \\ 0 & \alpha_2 & \dots & 0 \\ \vdots & \vdots & \ddots & \vdots \\ 0 & 0 & \dots & \alpha_k \end{bmatrix}$. Thus,

$$\mathbf{S} = \mathbf{X}\mathbf{Y}^{\mathrm{T}}\bar{\mathbf{Y}}^{\#} - \mathbf{W}_0\mathrm{Diag}(\boldsymbol{\alpha})\mathbf{H}_0\mathbf{Y}^{\mathrm{T}}\bar{\mathbf{Y}}^{\#}. \tag{S21}$$

The temporal complexity of forming S is $O(mnk + mk^2 + (m+n)r_0k + r_0k^2)$ and is dominated by $O(mnk + (m+n)r_0k)$.

Additional experimental results

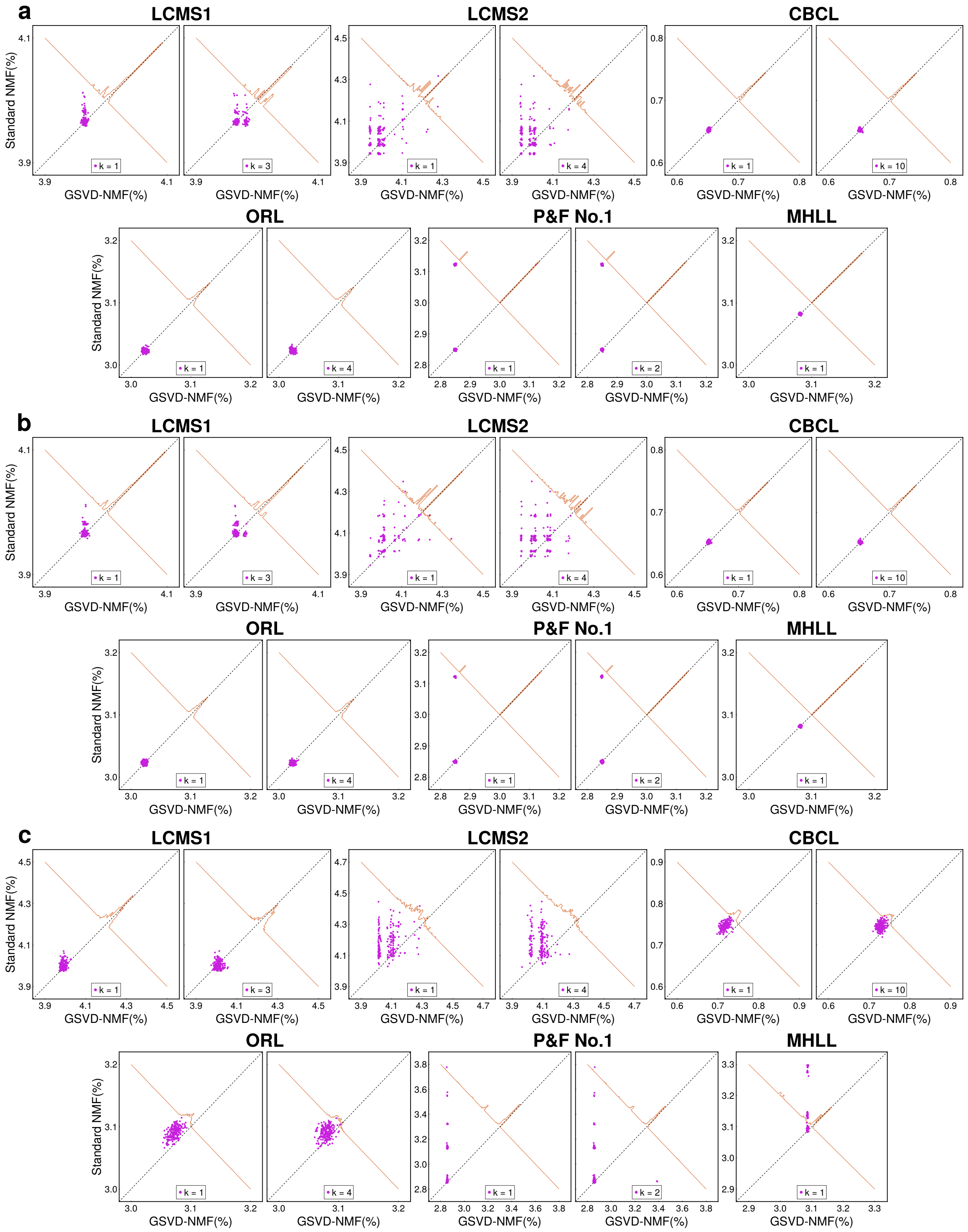

Figure S2: Comparing the fitting error of standard NMF (GCD, ALSGrad, MU) and GSVD-NMF on real-world data, related to Figure 5. (a) GCD. (b) ALSGrad. (c) MU. Note that the axes for MU are expanded compared to the other three algorithms.

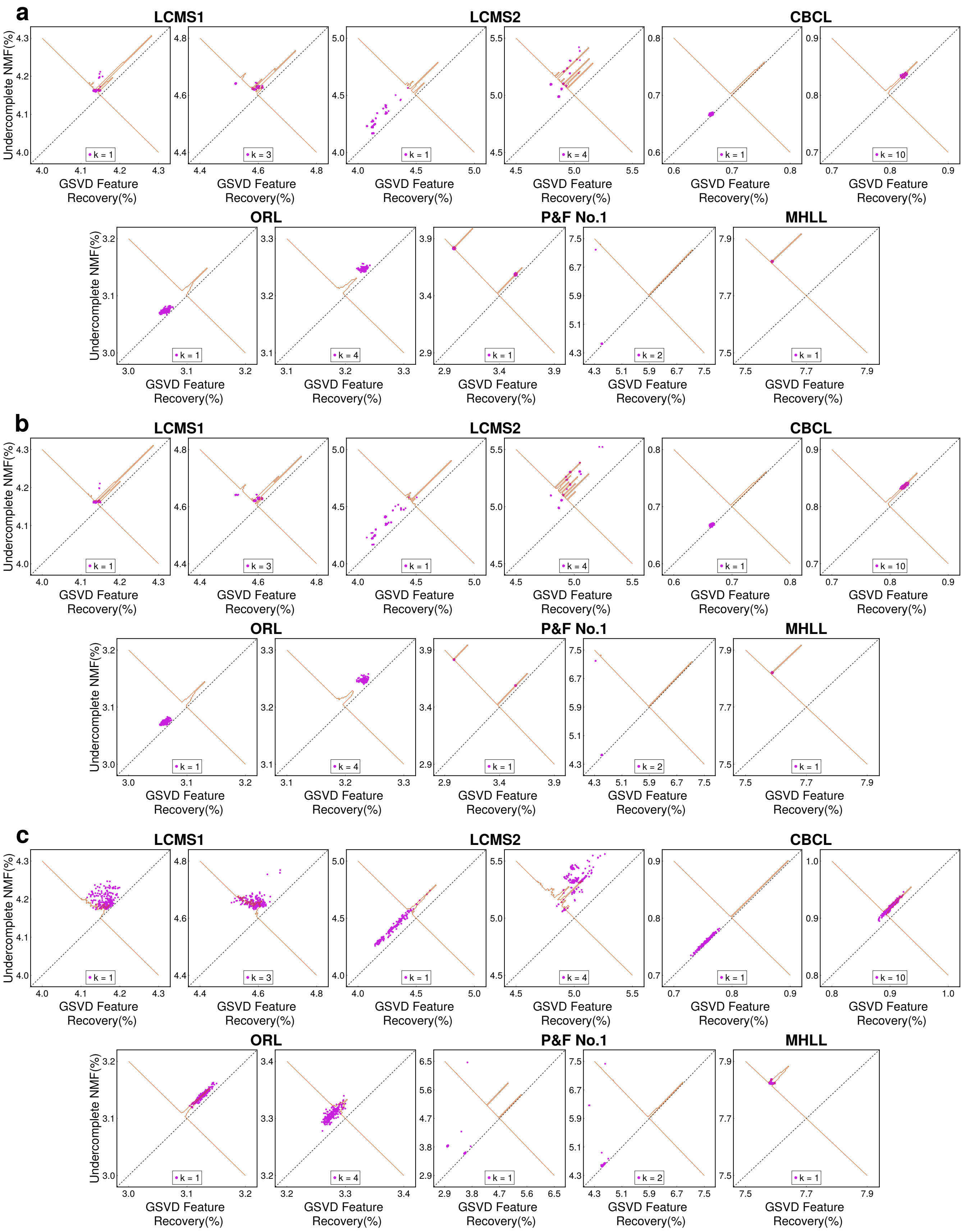

Figure S3: Comparing the fitting error of under-complete NMF (GCD, ALSGrad, MU) and GSVD feature recovery on real-world data, related to Figure 6. (a) GCD. (b) ALSGrad. (c) MU.

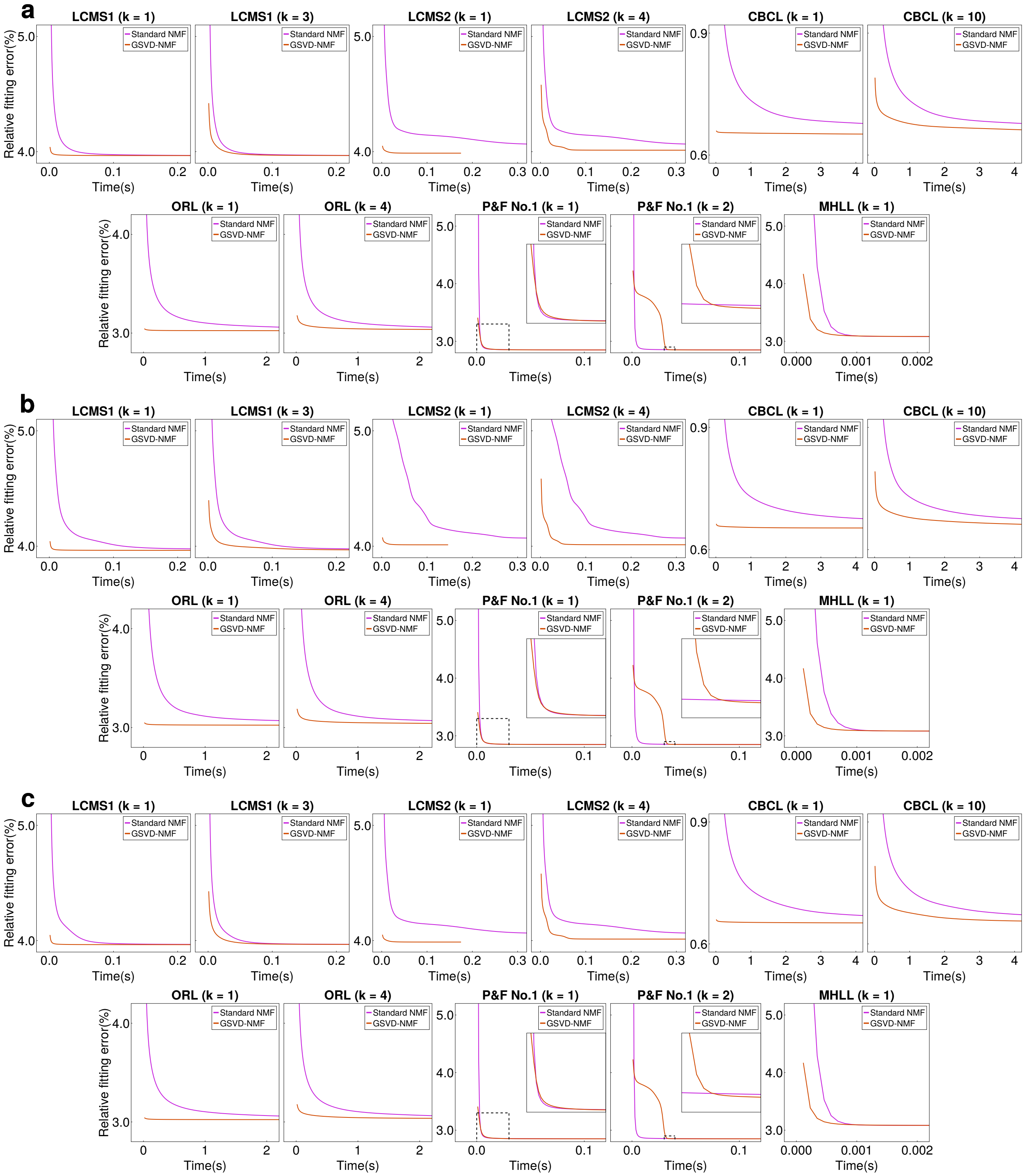

Figure S4: Runtimes of standard NMF vs GSVD-NMF with deterministic initialization, related to Figure 8. The plots show detailed convergence trajectories (objective value vs. time) during iteration for deterministic initialization. (a) NNDSVD. (b) NNDSVDa. (c) NNDSVDar. For most data sets and initializations, GSVD-NMF converged to high-quality solutions more quickly.

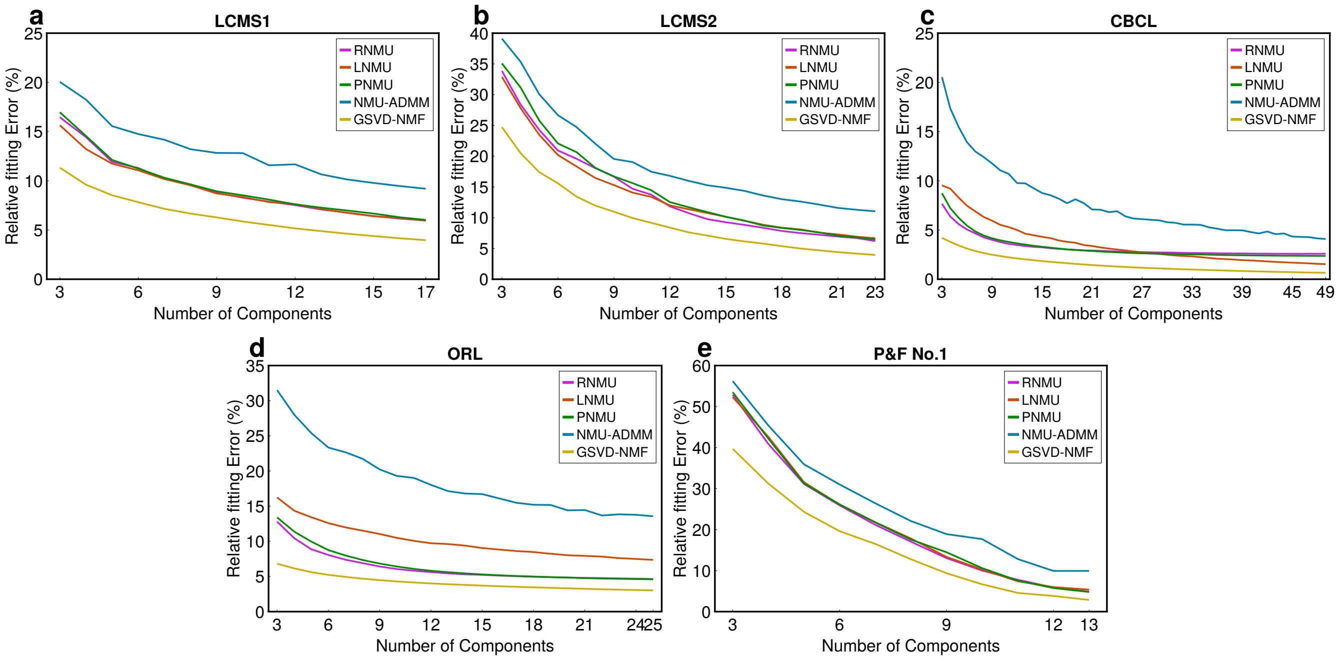

Figure S5: Comparison of the relative fitting error versus the number of components for GSVD-NMF (adding one component at a time) and NMU algorithms. The final fitting error at the end of each trajectory corresponds to the values reported in Table 3. (a) LCMS1. (b) LCMS2. (c) CBCL. (d) ORL. (e) Prelude and Fugue No.1 in C major.

Table S1: GSVD-NMF vs standard NMF (GCD, ALSPGrad, MU) with different initialization

	Fitting error (%) : Standard NMF / GSVD-NMF					
Data sets	r	k	Random	NNDSVD	NNDSVDa	NNDSVDar
GCD						
LCMS1	17	1	3.97±0.01 / 3.97±0.00	3.98 / 3.97	3.97 / 3.97	3.97 / 3.97
		3	3.97±0.01 / 3.97±0.01	3.98 / 3.97	3.97 / 3.97	3.97 / 3.97
LCMS2	23	1	**4.03±0.07 / 3.99±0.06**	**3.99 / 3.94**	4.00 / 4.10	**3.99 / 3.94**
		4	**4.03±0.07 / 3.98±0.04**	3.99 / 4.01	4.00 / 4.00	3.99 / 4.01
CBCL	49	1	0.65±0.00 / 0.65±0.00	0.65 / 0.65	0.65 / 0.65	0.65 / 0.65
		10	0.65±0.00 / 0.65±0.00	0.65 / 0.65	0.65 / 0.65	0.65 / 0.65
ORL	25	1	3.02±0.00 / 3.02±0.00	3.02 / 3.02	3.02 / 3.02	3.02 / 3.02
		4	3.02±0.00 / 3.02±0.00	3.02 / 3.03	3.02 / 3.02	3.02 / 3.02
P&F No.1	13	1	**2.90±0.11 / 2.85±0.00**	2.85 / 2.85	2.85 / 2.85	2.85 / 2.85
		2	**2.90±0.11 / 2.85±0.00**	2.85 / 2.85	2.85 / 2.85	2.85 / 2.85
MHLL	3	1	3.08±0.00 / 3.08±0.00	3.08 / 3.08	3.08 / 3.08	3.08 / 3.08
ALSPGrad						
LCMS1	17	1	3.97±0.01 / 3.96±0.00	3.97 / 3.97	3.96 / 3.97	3.97 / 3.97
		3	3.97±0.01 / 3.97±0.00	3.97 / 3.97	3.96 / 3.97	3.97 / 3.97
LCMS2	23	1	**4.06±0.06 / 4.02±0.04**	**3.99 / 3.94**	4.19 / 4.01	**3.99 / 3.94**
		4	**4.06±0.06 / 4.02±0.05**	3.99 / 4.01	**4.19 / 3.94**	3.99 / 4.01
CBCL	49	1	0.65±0.00 / 0.65±0.00	0.65 / 0.65	0.65 / 0.65	0.65 / 0.65
		10	0.65±0.00 / 0.65±0.00	0.65 / 0.65	0.65 / 0.65	0.65 / 0.65
ORL	25	1	3.02±0.00 / 3.02±0.00	3.02 / 3.02	3.02 / 3.02	3.02 / 3.02
		4	3.02±0.00 / 3.02±0.00	3.02 / 3.02	3.02 / 3.02	3.02 / 3.02
P&F No.1	13	1	**2.89±0.10 / 2.85±0.00**	2.85 / 2.85	2.85 / 2.85	2.85 / 2.85
		2	**2.89±0.10 / 2.85±0.00**	2.85 / 2.85	2.85 / 2.85	2.85 / 2.85
MHLL	3	1	3.08±0.00 / 3.08±0.00	3.08 / 3.08	3.08 / 3.08	3.08 / 3.08
MU						
LCMS1	17	1	**4.00±0.02 / 3.99±0.01**	**5.09 / 4.05**	**4.01 / 4.00**	**4.01 / 4.00**
		3	**4.00±0.02 / 4.01±0.01**	**5.09 / 4.04**	4.01 / 4.04	4.01 / 4.01
LCMS2	23	1	**4.18±0.08 / 4.08±0.06**	**5.89 / 4.23**	**4.43 / 4.31**	**4.08 / 3.99**
		4	**4.18±0.08 / 4.08±0.04**	**5.89 / 4.17**	**4.43 / 4.11**	**4.16 / 4.10**
CBCL	49	1	**0.75±0.01 / 0.72±0.01**	**1.66 / 0.81**	**0.79 / 0.75**	**0.76 / 0.73**
		10	**0.75±0.01 / 0.73±0.01**	**1.66 / 0.80**	**0.79 / 0.74**	**0.77 / 0.75**
ORL	25	1	**3.09±0.01 / 3.07±0.01**	**4.05 / 3.13**	**3.10 / 3.08**	**3.12 / 3.09**
		4	**3.09±0.01 / 3.08±0.01**	**4.05 / 3.12**	**3.10 / 3.09**	**3.12 / 3.09**
P&F No.1	13	1	**2.93±0.15 / 2.86±0.00**	**4.50 / 2.86**	2.86 / 2.86	2.86 / 2.86
		2	**2.93±0.15 / 2.86±0.05**	**4.50 / 2.86**	2.86 / 2.87	2.86 / 2.87
MHLL	3	1	**3.12±0.06 / 3.09±0.00**	**3.24 / 3.10**	3.09 / 3.09	3.09 / 3.09
